# Habitat Shifts and Conservation Challenges of Falconidae Under Climate Change in Northwestern China

**DOI:** 10.3390/ani16172650

**Published:** 2026-08-24

**Authors:** Shugao Wang, Xuejun Ma, Jiejun Li, Hongshan Li, Xi Jin, Xiaoling Zhang, Ying Zhao, Ning Li, Feng Xu

**Affiliations:** 1 College of Life Science, Shihezi University, Shihezi 832003, China; 2 State Key Laboratory of Ecological Safety and Sustainable Development in Arid Lands, Xinjiang Institute of Ecology and Geography, Chinese Academy of Sciences, Urumqi 830011, China; 3 China-Tajikistan Belt and Road Joint Laboratory on Biodiversity Conservation and Sustainable Use, Xinjiang Institute of Ecology and Geography, Chinese Academy of Sciences, Urumqi 830011, China; 4 Forestry and Grassland Resources Monitoring Center, Xinjiang Production and Construction Corps, Urumqi 830013, China; 5 Center for Wildlife Conservation and Monitoring of Xinjiang Uygur Autonomous Region, Urumqi 830000, China; 6 Institute of Applied Ecology, Nanjing Xiaozhuang University, Nanjing 211171, China

**Keywords:** falconidae, species distribution modeling, MaxEnt, InVEST, climate change, habitat suitability, protected areas, barycenter migration, Xinjiang, raptors

## Abstract

Xinjiang’s distinctive geography and climate provide important habitats for Falconidae species, yet their climate-driven habitat shifts and conservation gaps remain poorly understood. Using 2731 validated occurrence records and 26 environmental predictors, including bioclimatic, land-use, topographic, hydrological, anthropogenic, and Normalized Difference Vegetation Index (NDVI) predictors, we applied an optimized Maximum Entropy (MaxEnt) framework to project suitable habitats for seven falconid species under current conditions and three Shared Socioeconomic Pathway scenarios (1–2.6, 2–4.5, and 5–8.5) for 2041–2060, 2061–2080 and 2081–2100. Barycenter migration analysis, the Habitat Quality module of the Integrated Valuation of Ecosystem Services and Tradeoffs framework, and protected-area overlays were further integrated to identify conservation priorities. All models showed high discriminatory performance, with mean areas under the receiver operating characteristic curve exceeding 0.90. Current suitable habitats were mainly concentrated along river corridors and mountain foothills in the southern Altai, central-western Tianshan and northern Kunlun regions. Future responses were strongly species-specific. By 2081–2100 under Shared Socioeconomic Pathway 5–8.5, suitable habitat increased by 154.6% for *Falco peregrinus* and 79.5% for *Falco tinnunculus* but declined by 81.1% for *Falco vespertinus*; *Falco subbuteo* also showed overall expansion, with its suitable-habitat barycenter shifting by up to 291.9 km. Mean relative habitat quality was 0.514 [standard deviation = 0.185], while suitable habitat outside protected areas ranged from 35,346 to 304,866 km^2^ among species, and high-quality priority conservation gaps reached 102,489 km^2^ for *Falco cherrug*. These results demonstrate that climatic suitability does not necessarily correspond to high habitat quality or adequate protection and support species-specific, climate-adaptive conservation strategies for falconids in Xinjiang.

## Simple Summary

Climate change and land-use shifts threaten falcons in Xinjiang, China. Using over 2700 occurrence records and environmental data, we modeled current and future habitats for seven species under multiple climate scenarios and assessed habitat quality and protected-area coverage. Suitable habitats now concentrate in mountain foothills and river valleys. Future responses are species-specific: Common Kestrel and Peregrine Falcon may expand, whereas Red-footed Falcon and Saker Falcon are projected to decline sharply under severe warming. Importantly, many climatically suitable areas lie outside protected reserves and face high degradation risk, meaning suitability does not guarantee effective protection. We recommend expanding protected areas, restoring degraded habitats, and maintaining corridors to support climate-adaptive conservation.

## 1. Introduction

The reciprocal interaction between species and their environment is a central ecological process governing ecosystem structure, function, and stability [1]. In the Anthropocene, habitat loss, fragmentation, pollution, biological invasions, and climate change are disrupting these relationships at rates that may exceed the adaptive capacity of many species [2,3,4,5]. Among terrestrial vertebrates, birds are especially responsive to such environmental change because climatic and habitat conditions strongly influence their distributions, phenology, and population dynamics [6].

Birds are highly responsive to environmental change because their geographic distributions, migration, breeding phenology, and population dynamics are closely linked to climatic and habitat conditions. Changes in temperature and precipitation can directly alter physiological and energetic constraints and can indirectly affect vegetation phenology, food availability, and the timing of migration and reproduction. Consequently, climate change may cause range expansion or contraction, latitudinal and elevational redistribution, and shifts in the geographic centers of suitable habitats [7]. In China, future climate change is projected to alter the extent and distributional centroids of suitable habitats for migratory birds, while long-term observations indicate that climatic conditions can also modify the migration timing of raptors [8,9]. These responses make birds an informative group for evaluating the ecological consequences of climate change.

Raptors are particularly informative for evaluating environmental change because they occupy high trophic positions and depend on the availability of prey, suitable nesting substrates, and functionally connected landscapes. Their generally large spatial requirements, long lifespans, delayed maturity, and low reproductive rates may limit their capacity to compensate rapidly for habitat loss, increased disturbance, or additional mortality. At the global scale, raptors are more threatened than birds as a whole, and many raptor populations are declining under the combined effects of habitat alteration, agricultural expansion, infrastructure development, and other human pressures [10,11]. Among the raptors occurring in Xinjiang, many Falco species use open or semi-open landscapes, including grasslands, desert steppes, agricultural mosaics, river valleys, and mountain foothills. Their habitat use is therefore likely to respond jointly to climate, topography, vegetation structure, water availability, land-use change, and human disturbance. Differences in migration strategy, prey use, nesting requirements, and habitat specialization may further generate species-specific responses to future environmental change [12,13]. Studies from Southeast Asia further illustrate that raptor habitat use and climate responses are strongly influenced by local habitat conditions. In Peninsular Malaysia, resident Peregrine Falcons (*Falco peregrinus ernesti*) breed mainly on limestone cliffs, highlighting their dependence on specific nesting substrates and potential exposure to land-use disturbance [14]. In Indonesia, ecological niche modeling of the Javan Hawk-Eagle (*Nisaetus bartelsi*) projected substantial future habitat contraction under the combined effects of climate change and anthropogenic pressure [15]. These findings emphasize the importance of considering climatic suitability together with habitat condition and human disturbance in raptor conservation.

Xinjiang provides an appropriate regional setting for examining these processes because its mountain-basin topography creates pronounced spatial gradients in temperature, precipitation, vegetation, water availability, and human land use. As a representative arid and semi-arid region of western China, Xinjiang contains diverse but environmentally fragile ecosystems that are highly sensitive to climatic change [16]. The region supports considerable avian diversity, with 425 recorded bird species and 52 raptor species [17]. However, previous studies of raptor distributions in Xinjiang have generally been limited by incomplete occurrence records, restricted spatial coverage, or insufficient consideration of future climate change [12,18,19,20,21]. More importantly, predicted environmental suitability does not necessarily correspond to high habitat quality or adequate protection, yet these dimensions have rarely been evaluated together for Falconidae in the region [22]. In addition, species-specific differences in future habitat change and spatial redistribution remain poorly understood. These gaps limit the identification of conservation priorities and the development of species-specific, climate-adaptive conservation strategies.

Falconid distributions are jointly constrained by climatic, topographic, hydrological, and landscape processes [13]. Temperature and precipitation can affect thermoregulatory and energetic costs, breeding and migration phenology, vegetation productivity, and the abundance or accessibility of prey [23]. Elevation, slope, and aspect modify local temperature and moisture conditions, terrain structure, and the availability of nesting and foraging sites [24]. Land-use/land-cover and NDVI characterize landscape composition, vegetation productivity, and vegetation structure, which may influence prey resources as well as hunting efficiency in open and semi-open environments [25,26]. Proximity to water may be particularly important in arid regions because river valleys, wetlands, reservoirs, and oases concentrate vegetation productivity and potential prey. Human land use and infrastructure can further alter nesting opportunities, disturbance exposure, mortality risk, and landscape connectivity [27]. These ecological mechanisms provide the biological basis for integrating climatic, topographic, vegetation, hydrological, and land-use predictors when modeling falconid habitat suitability. The MaxEnt model is based on the principle of maximum entropy and estimates a distribution of relative habitat suitability across the study area that maximizes entropy subject to constraints derived from species occurrence records and environmental predictors [28,29]. It is widely used for presence-only data and can perform effectively with relatively limited occurrence records, provided that sampling bias and model complexity are carefully addressed. However, default parameter settings may generate unnecessarily complex models and reduce transferability. Species-specific optimization of feature classes and regularization multipliers can therefore reduce overfitting and improve predictive performance [30,31]. Changes in projected suitable-habitat extent and distributional barycenters can provide spatial indicators of potential habitat redistribution under alternative future climate and land-use scenarios [32]. Importantly, shifts in modeled habitat barycenters reflect the spatial redistribution of predicted environmental suitability rather than direct observations of individual movement, dispersal, or migration.

However, environmental suitability predicted by species distribution models does not necessarily correspond to intact or high-quality habitat conditions on the ground. Areas identified as suitable by species distribution models (SDMs) may still be affected by habitat degradation, fragmentation, intensive land-use pressure, or other anthropogenic disturbances, which may reduce their potential conservation value despite otherwise favorable climatic or environmental conditions [13,22]. Therefore, integrating modeled habitat suitability, relative habitat quality, degradation pressure, and protected-area representation can provide a more reliable basis for identifying conservation priorities. In this context, the InVEST Habitat Quality model provides a spatially explicit, land-cover-based assessment of relative habitat condition and degradation pressure. It can therefore complement MaxEnt-based suitability predictions by distinguishing environmentally suitable areas that remain relatively intact from those exposed to substantial anthropogenic disturbance [33,34,35].

In addition, protected areas are central instruments for biodiversity conservation, but their effectiveness depends not only on their total area but also on whether they adequately cover the most important habitats of target species [36,37]. Conservation gap analysis, which overlays species distribution or habitat suitability maps with protected-area boundaries, has been widely used to assess whether priority habitats are sufficiently represented within existing conservation networks and to identify unprotected or underrepresented areas requiring additional conservation attention [38]. For raptors with large home ranges and high sensitivity to habitat disturbance, such spatial overlay analyses are particularly valuable because suitable habitats may extend beyond formal protected areas or occur in landscapes with high degradation risk [39,40]. To address these knowledge gaps, we integrated an optimized MaxEnt framework with barycenter migration analysis, InVEST-based habitat assessment, and protected-area overlay analysis to evaluate current habitat patterns, future redistribution, habitat conditions, and conservation gaps for Falconidae in Xinjiang [28,29,30,41,42]. Accordingly, this study integrates optimized habitat-suitability modeling, barycenter analysis, habitat quality and degradation assessment, and protected-area overlays to move beyond suitability-only evaluations. Specifically, we address the following questions: (1) What are the current spatial patterns of suitable habitats? (2) How will suitable habitats change under future climate scenarios? (3) How will suitable-habitat barycenters shift? (4) Where are the main conservation gaps? (5) How do future responses differ among species? By linking habitat suitability, future redistribution, habitat condition, and protection status, this study provides a basis for species-specific and climate-adaptive conservation in Xinjiang.

## 2. Materials and Methods

### 2.1. Study Area

**Figure 1:**
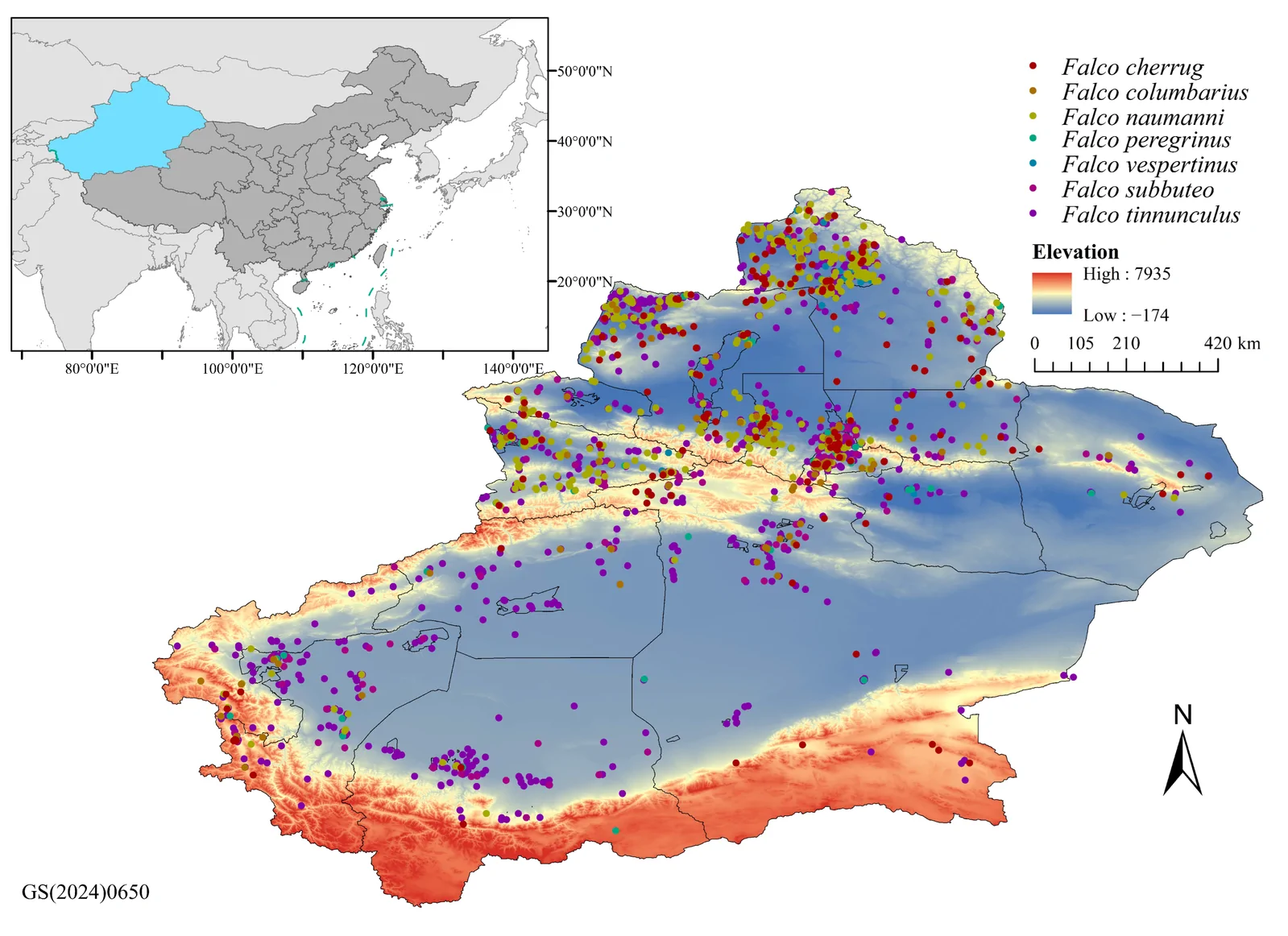
Geographic location, elevation, and validated occurrence records of seven falconid species in Xinjiang, China. The main map shows the elevation of the study area and species occurrence records, and the inset shows the location of Xinjiang within China.

The Xinjiang Uygur Autonomous Region (34°22′–49°33′ N, 73°41′–96°18′ E) covers approximately 1.6649 million km^2^ in northwestern China and is a typical inland arid-semi-arid region of Eurasia [43]. Its topography is characterized by “three mountain ranges separated by two basins,” comprising, from north to south, the Altai Mountains, Junggar Basin, Tianshan Mountains, Tarim Basin, and Kunlun Mountains. This mountain-basin system includes plateaus, high mountains, hills, and basins and creates pronounced climatic gradients [44]. The Tianshan Mountains act as a major geographic barrier that modulates moisture transport by the westerlies and blocks or channels northerly cold air, concentrating precipitation in northern valleys such as the Ili and Irtysh and producing marked north-south differences in temperature and precipitation [45]. Xinjiang is characterized by an arid continental climate, with limited precipitation, high evaporation, and scarce water resources. Its sparse vegetation, low ecosystem productivity, strong dependence on river runoff and mountain snowmelt, and slow recovery after disturbance make regional ecosystems particularly vulnerable to climate change [17]. Recent warming and humidification trends may further intensify hydrological processes and challenge regional ecological stability [18]. The geographic location, elevation, and occurrence records of the seven falconid species in the study area are shown in Figure 1.

### 2.2. Data Sources

#### 2.2.1. Bird Occurrence Data

We initially selected nine Falconidae species recorded as occurring in Xinjiang according to A Checklist on the Classification and Distribution of the Birds of China (Fourth Edition) [46]: Amur Falcon (*Falco amurensis*), Saker Falcon (*F. cherrug*), Merlin (*F. columbarius*), Lesser Kestrel (*F. naumanni*), Peregrine Falcon (*F. peregrinus*), Gyrfalcon (*F. rusticolus*), Eurasian Hobby (*F. subbuteo*), Common Kestrel (*F. tinnunculus*), and Red-footed Falcon (*F. vespertinus*). The same checklist was also used as the taxonomic reference for species identification and scientific nomenclature throughout this study.

In total, 10,498 raw occurrence records were assembled [47,48,49] from three sources: (1) 150 falconid records from field surveys across Xinjiang conducted from August 2023 to October 2025, covering reservoirs, lakes, nature reserves, and ecological parks, during which observers recorded species identity, abundance, and geographic coordinates while traveling transects by vehicle (20–40 km/h); (2) 9037 records from citizen science and institutional observations for Xinjiang (January 2002–July 2025) retrieved from the China Bird Report Center (https://www.birdreport.cn) via automated data acquisition using Selenium (version 4.47.0) in Python (version 3.10.11) after cleaning; and (3) 1311 occurrences downloaded from Global Biodiversity Information Facility (GBIF) (https://www.gbif.org) for the same period [50,51,52,53,54,55,56,57,58].

To reduce spatial autocorrelation and sampling bias, the records were first grouped by species and converted into CSV files containing species names and decimal WGS84 coordinates. We performed spatial thinning using the thin() function in the spThin package (version 0.2.0) in R (version 4.5.2) with a 1 km thinning distance, thereby retaining at most one occurrence per 1 km × 1 km grid cell. This resolution was chosen to minimize spatial autocorrelation while aligning with the highest resolution of our environmental predictor variables [59,60]. To ensure adequate sample support for species distribution modeling, only species with ≥100 unique occurrence records after spatial thinning were retained for subsequent analyses [20,61,62,63].

#### 2.2.2. Environmental Variables

Environmental predictors comprised three categories: (1) natural habitat factors, including distance to water bodies, distance to nature reserves, land use and land cover change (LUCC), and normalized difference vegetation index (NDVI); (2) topographic factors, including elevation, slope, and aspect; and (3) climatic factors, including 19 bioclimatic variables.

The distance-to-water and distance-to-nature-reserve layers were derived from hydrographic and reserve boundary data from the 1:1,000,000 National Fundamental Geographic Database (National Geomatics Center of China (https://www.webmap.cn/commres.do?method=result100W, accessed on 18 August 2026)) and the Resource and Environmental Science and Data Platform (https://www.resdc.cn), respectively. Distance rasters were computed using the “Near” analysis in ArcGIS 10.8 (Esri, Redlands, CA, USA) [64].

Current LUCC data (2020) were obtained from the National Geomatics Center of China (https://cloudcenter.tianditu.gov.cn, accessed on 18 August 2026) global land-cover dataset [65]; and future LUCC data (1-km resolution) were obtained from the dataset of Zhang et al. [66] (GCAM+PLUS outputs). To maintain consistency with the climate projections used in this study, only the SSP1-2.6, SSP2-4.5, and SSP5-8.5 scenarios from the original dataset were included in the modeling analyses [66]. NDVI: annual 1-km NDVI (2002–2023) from the Resource and Environmental Science Data Center (https://www.resdc.cn); multi-year mean values were calculated as a static vegetation indicator [67].

Topography: ASTER GDEM (30-m) provided elevation, from which slope and aspect were derived (https://www.gscloud.cn).

Climate: present-day climate used WorldClim v2.1 bioclimatic variables (1970–2000 baseline) (https://www.worldclim.org). Future climate projections were made using the CMIP6 BCC-CSM2-MR model [68,69] under SSP126, SSP245, and SSP585 for three time slices (2041–2060, 2061–2080, and 2081–2100).

All raster layers were resampled to 1-km spatial resolution, reprojected to WGS84, and masked to the administrative boundary of Xinjiang (sourced from the National Geomatics Center of China). The final rasters were exported in ASCII format as input to MaxEnt V3.4.4. (Table 1).

### 2.3. Modeling Procedures

#### 2.3.1. Model Construction and Variable Selection

We used MaxEnt version 3.4.4 to model the current and future habitat suitability of each retained Falconidae species. Predictor selection and model-complexity optimization were conducted separately for each species using the following procedures. We employed a two-stage process to select the variables, minimizing multicollinearity and overfitting. First, Spearman’s rank correlation coefficients between all candidate predictors were computed using background points (SDMtune package (version 1.3.3) in R). Variable pairs with |ρ| > 0.8 were considered highly correlated; in such cases, we retained the variable with greater ecological relevance for falconids or higher preliminary contribution to the model performance and removed the other variable. Second, we performed stepwise variable elimination based on the model discrimination performance, in which variables were iteratively removed and the resulting model AUC on an independent test set was recorded. The subset of variables that yielded the highest AUC and meaningful ecological contributions was retained. We also used the Jackknife test within MaxEnt to quantify the importance of individual variables and identify redundant variables [70]. The final species-specific predictor subsets obtained through this procedure were used for subsequent MaxEnt calibration.

In the MaxEnt model, the two parameters that most strongly affect the simulation results are feature classes (FCs) and the regularization multiplier (RM). The RM value determines the model complexity, whereas the FC determines the potential shape of the marginal response curves. MaxEnt provides five feature classes: linear (L), quadratic (Q), product (P), threshold (T), and hinge features (H). The more feature types selected, the more complex the model becomes [71]. Therefore, different parameter combinations produce models with different levels of complexity. To avoid overfitting and improve the generalization ability, we used the ENMeval package (version 2.0.5.2) in R to systematically optimize the key hyperparameters of the MaxEnt model. Eight feature-class combinations (FCs), namely L, LQ, LQH, LQHP, LQHPT, QHP, QHPT, and HPT, were combined with eight regularization multipliers (0.5–4.0, with an interval of 0.5), generating 64 parameter sets for analysis. The optimal model parameters were selected based on the ΔAICc (the minimum information criterion AICc value, ΔAICc = AICc_i_ − AICcmin), and when the ΔAICc value was minimal, model complexity and predictive performance were considered optimal [72].

The optimized environmental variables and parameter settings were imported into MaxEnt (V3.4.4) for final modeling. Response curves were generated, and a jackknife test was used to evaluate the contribution of environmental variables. The output format was set to “logistic”. To characterize environmental availability, 10,000 background points were randomly sampled from the entire study area. While this approach provides a broad representation of the available conditions, potential interactions with species occurrence sampling bias (as discussed later) are acknowledged. Of the occurrence data, 25% were randomly selected as the validation dataset for model evaluation, and the remaining 75% were used as training datasets. This cross-validation procedure was repeated 10 times, with the replicated run type set to “Crossvalidate,” whereas all other parameters were maintained at their default settings. The corresponding future environmental variables were then incorporated into the analyses under future climate scenarios, yielding predictions of current and future suitable habitats for each species [73].

The ASCII-format distribution maps generated by the model were reclassified in ArcGIS 10.8. The “10 percentile training presence Logistic threshold” was used to distinguish suitable from unsuitable habitats, as this threshold is commonly employed to balance omission and commission errors and is considered robust for identifying areas with a high probability of species presence. Suitable habitats were further divided into low-, medium-, and high-suitability classes using the tertile classification method. The areas of suitable and unsuitable habitats under different periods and climate scenarios were calculated using the Raster Calculator [74,75,76].

#### 2.3.2. Barycenter Migration of Suitable Habitats of Falconid Birds

Climate change may alter the spatial distribution of suitable habitats and drive species to shift toward areas with more favorable environmental conditions. To characterize these spatial changes, the barycenter, defined as the geographic center of the predicted suitable habitat, was used as an indicator of habitat redistribution. By comparing the location and movement of the barycenter among different periods and climate scenarios, changes in the direction and distance of suitable-habitat shifts can be quantified, thereby providing a spatial measure of potential habitat migration patterns under climate change [8,62,77].

To calculate the barycenter (distribution center) of suitable habitats, we first extracted the suitable habitat areas for each species using the Extract by Attributes tool. These raster layers were converted into polygon features using the Raster to Polygon tool. Finally, the Mean Center spatial statistics tool was used to utilize the spatial geometric information of these polygon features to calculate the distribution center. For the multi-period results, barycenter points from different periods were merged, whereas the scenario and period fields were retained to support subsequent grouping and ordering. In ArcGIS 10.8, the merged barycenter points were connected in chronological order to generate migration trajectories using Points to Line. During trajectory construction, the scenario was used as the grouping field (Line Field) and the period as the sorting field (Sort Field), thereby ensuring that barycenter points under the same scenario were correctly connected according to the temporal sequence. This yielded barycenter migration paths for each scenario and enabled spatial visualization [78,79,80,81].

To extract elevation information for the barycenter points and quantitatively calculate their relative changes, while also uniformly outputting migration distance and direction relative to the current period, batch calculations were conducted in the R environment. A digital elevation model (DEM; ASCII raster format, .asc) was read using the terra (version 1.8.80) package, and elevation values were extracted for each barycenter point through raster-point spatial overlay to obtain barycenter elevations for all periods. Subsequently, using the barycenter of the current period (Current) as the common reference, the elevation change for each future period was calculated as ΔElev = Elev_future − Elev_current, and the sign of this value was used to determine whether the elevation increased or decreased. The barycenter migration distance was calculated using the geodesic distance function distGeo() provided by the geosphere (version 1.5.20) package, which converts the spherical/ellipsoidal distance between the current barycenter and each future barycenter into kilometers, thereby avoiding the systematic errors associated with planar distance calculations under geographic coordinates. The migration direction was calculated using geosphere::bearing(), which derives the azimuth (bearing; 0° = true north, increasing clockwise) from the reference point to the target point, and was further classified into eight compass directions (north, northeast, east, southeast, south, southwest, west, and northwest) to facilitate result summarization and comparison.

#### 2.3.3. InVEST-Based Habitat Quality and Degradation Assessment

We used the InVEST Habitat Quality model (version 3.3.2) to assess landscape-based relative habitat quality and habitat degradation risk relevant to Falconidae conservation in Xinjiang. The model estimates habitat degradation and habitat quality by integrating land-use/land-cover (LULC) types, spatial layers of threat factors, threat weights, maximum distances of influence, distance-decay functions, and the sensitivity of each LULC type to different threats [35]. In this study, the InVEST outputs were interpreted as relative habitat conditions derived from land-cover patterns and threat intensity, rather than species-specific realized habitat quality, actual occupancy probability, population density, or absolute ecological thresholds. These outputs were further used in combination with MaxEnt-derived habitat suitability maps to identify areas that were both environmentally suitable and characterized by relatively high current habitat quality [34,36]. The model was run using a 30 m LULC raster, and the LULC classes within the study area were reclassified into cropland, forest, grassland, shrubland, wetland, water, artificial surface, bare land, and glacier/permanent snow. All threat layers were projected, rasterized, resampled, and aligned to the same spatial resolution, extent, coordinate system, and pixel grid as the LULC raster. Parameterization followed the requirements of the Habitat Quality module in the InVEST User’s Guide and was further adjusted within the ranges of maximum influence distance, threat weight, and distance-decay function reported in previous habitat quality studies [82,83,84]. Based on the landscape characteristics of Xinjiang and the habitat ecology of falconids, four threat factors were included: transportation disturbance, artificial surfaces, cropland, and bare land [85,86]. Roads and railways were merged to represent transportation disturbance, whereas artificial surfaces, cropland, and bare land represented construction-related disturbance, agricultural disturbance, and low-vegetation or exposed-surface disturbance, respectively. Transportation disturbance and cropland were assigned linear decay functions to represent a gradual decrease in influence with increasing distance, whereas artificial surfaces were assigned an exponential decay function to reflect stronger near-source disturbance and rapid attenuation with distance [87,88]. The full threat-factor parameters and their ecological interpretations are provided in Table 2.

Habitat suitability scores and threat sensitivity values for each LULC type were assigned according to the InVEST parameter framework, the ecological attributes of land-cover types in Xinjiang, and falconid use of open habitats, piedmont zones, river valleys, desert steppe, and cropland-grassland mosaic landscapes (Table 3). Grassland was assigned a relatively high habitat suitability score because it represents an important open foraging habitat for many falconid species in Xinjiang [89]. Shrubland, wetland, forest, and cropland were assigned moderate or moderate-to-low suitability values because they may provide prey resources or activity space in piedmont areas, river valleys, oasis margins, forest edges, or agricultural mosaic landscapes [27,90]. Water bodies were assigned low suitability because water itself is not a primary habitat for falconids, although riparian and wetland margins may have ecological relevance. Artificial surfaces were considered non-natural habitats and were assigned a habitat suitability score of 0. Bare land was not treated as unsuitable habitat because bare land in Xinjiang includes natural open landscapes such as Gobi, desert, exposed rock, and gravel surfaces, some of which may be used by falconids for foraging, resting, or breeding [26]. Therefore, bare land retained a certain habitat value in the sensitivity table, while being assigned a relatively low threat weight when used as a threat source to avoid overestimating degradation across extensive natural open landscapes. The resulting habitat quality and degradation maps were used for relative conservation prioritization and subsequent overlay analyses with MaxEnt suitability maps and protected-area boundaries; the derived classes indicate relative spatial priority and should not be interpreted as absolute ecological thresholds.

#### 2.3.4. Protected-Area Overlay and Conservation Gap Analysis

To evaluate the extent to which the existing protected-area system covers key habitats for Falconidae in Xinjiang, we extracted 82 protected areas within the Xinjiang Uygur Autonomous Region from the protected-area spatial dataset, including nature reserves, wetland parks, geoparks, forest parks, and scenic areas. The vector boundaries of these protected areas were converted into a binary raster layer, in which pixels located inside protected-area boundaries were assigned a value of 1 and pixels outside protected-area boundaries were assigned a value of 0. The resulting protected-area layer was then aligned with the MaxEnt-derived habitat suitability maps, InVEST-derived habitat quality map, and habitat degradation map using the same projected coordinate system, spatial extent, pixel size, and raster grid alignment.

By overlaying the protected-area layer with habitat quality and degradation classes, we calculated the area and proportion of each habitat quality and degradation class inside and outside protected areas to evaluate the representativeness of the existing protected-area system for key habitat categories [37,38]. Furthermore, for each falconid species, the continuous MaxEnt suitability output was converted into a binary suitable/unsuitable habitat map using the 10th percentile training presence logistic threshold, with suitable habitat including low-, medium-, and high-suitability areas. The binary suitable habitat map was then overlaid with the protected-area layer and the InVEST-derived habitat quality and degradation classes. Suitable habitats located outside protected areas were defined as potential conservation gaps and were further characterized according to their relative habitat quality and degradation risk. Suitable habitats outside protected areas with high relative habitat quality were considered priority conservation gaps, whereas those with low relative habitat quality or high degradation risk were regarded as potential management gaps requiring field validation, habitat restoration, or disturbance control [29,34,81].

The attribute-based conservation-gap categories were calculated independently and were therefore non-mutually exclusive. Consequently, the areas of individual gap categories should not be summed to estimate the total conservation-gap area. For cartographic presentation, a mutually exclusive gap map was generated by assigning each overlapping pixel to a single category according to a predefined priority hierarchy.

## 3. Results

### 3.1. Occurrence-Data Filtering, Predictor Selection, and Model Evaluation

Spatial thinning retained 2736 independent occurrence records. *Falco amurensis* (*n* = 2) and *F. rusticolus* (*n* = 3) did not meet the minimum occurrence threshold and were therefore excluded. Consequently, seven species comprising 2731 occurrence records were retained for MaxEnt modeling (Table 4).

Predictor screening generated species-specific environmental-variable subsets. BIO2, BIO3, BIO15, LUCC, distance to water bodies, distance to protected areas, and NDVI were retained for all seven modeled species, whereas the remaining predictors were retained selectively according to their correlation structure, ecological relevance, and model performance (Table 5).

ENMeval-based tuning produced species-specific optimal combinations of regularization multipliers (RMs) and feature classes (FCs) that substantially reduced the model complexity relative to the default settings (Table 6). For six species, the optimized models achieved ΔAICc = 0 within the tested parameter space, indicating markedly lower AICc than the defaults and suggesting an improved balance between model fit and complexity relative to the default settings. The selected RM-FC combinations were used to generate current and future habitat projections for each species in the study.

**Figure 2:**
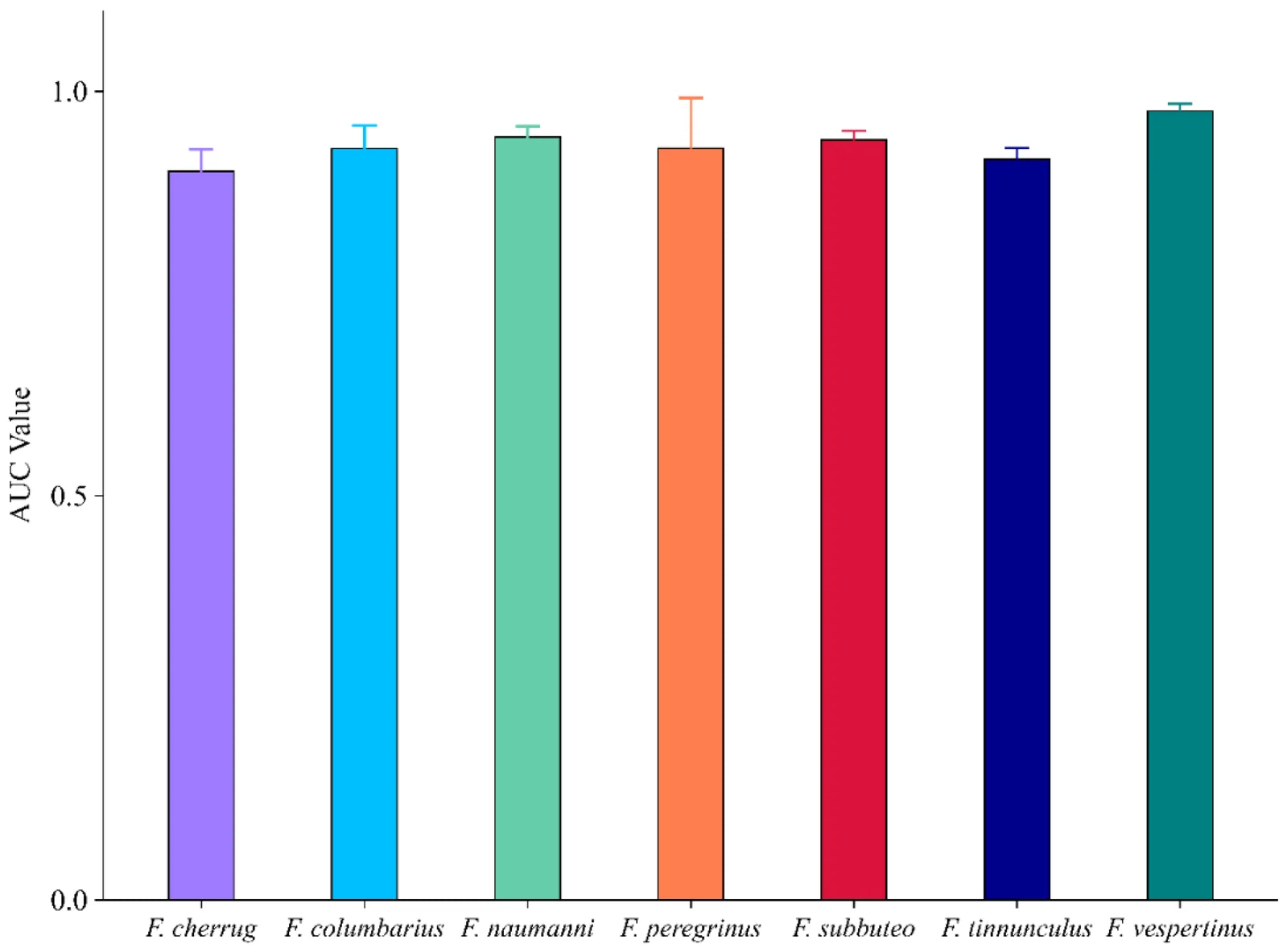
Simulated AUC values of Falconidae species in Xinjiang based on the MaxEnt model.

Model discrimination was high, with all species achieving mean AUC values exceeding 0.90 across current and future scenarios (standard deviation < 0.07; Figure 2). Further, inspection of avg.diff.AUC and Mean.OR10% confirmed acceptable training-testing consistency and omission rates (Table 4). These robust diagnostic metrics collectively support the reliability of the optimized MaxEnt models for subsequent spatial analyses.

### 3.2. Environmental Contributions and Response Curves

The percent contribution and jackknife analyses revealed marked interspecific differences in the key drivers, although some common patterns emerged (Table 7). LUCC (land-use/land-cover) was a dominant contributor for six species (14.3–76.1%), indicating the broad sensitivity of falconids to landscape composition. Precipitation variables, particularly BIO19 (precipitation of the coldest quarter) and BIO15 (precipitation seasonality), were important for several species, emphasizing the role of winter hydrothermal conditions. NDVI notably influenced *F. subbuteo* (29.7%), whereas distance to water bodies (Dis_ws) and proximity to protected areas (Dis_nr) also contributed to the presence of specific taxa.

Response curves revealed marked interspecific differences in the associations between environmental predictors and habitat suitability. Predicted occurrence probability was generally highest for artificial surfaces, followed by water bodies, wetlands, or cropland (Figure S1). For NDVI, predicted suitability increased markedly once NDVI exceeded approximately 0.2. *Falco tinnunculus*, *F. cherrug*, and *F. subbuteo* approached plateaus at moderate NDVI values, whereas *F. columbarius*, *F. vespertinus*, and *F. peregrinus* peaked at approximately 0.5 and declined at higher values. *Falco naumanni* showed a renewed increase at high NDVI values (Figure S2). For climatic predictors, *F. tinnunculus*, *F. naumanni*, and *F. cherrug* exhibited unimodal responses to BIO19, with maxima at approximately 27–50 mm. By contrast, the predicted suitability of *F. vespertinus* declined rapidly when BIO15 exceeded approximately 25 (Figures S3 and S4).

### 3.3. Current Spatial Patterns of Suitable Habitat

**Figure 3:**
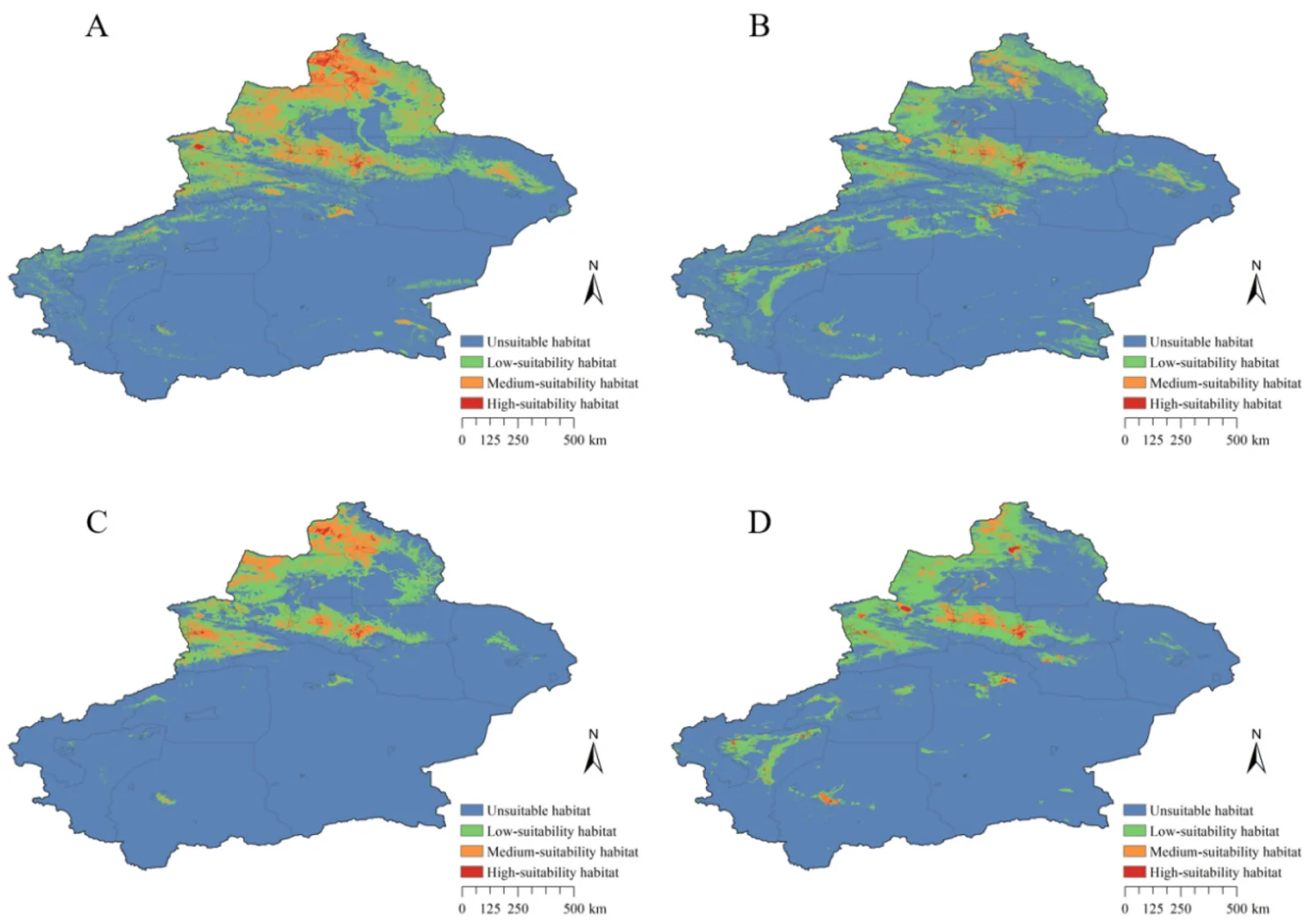

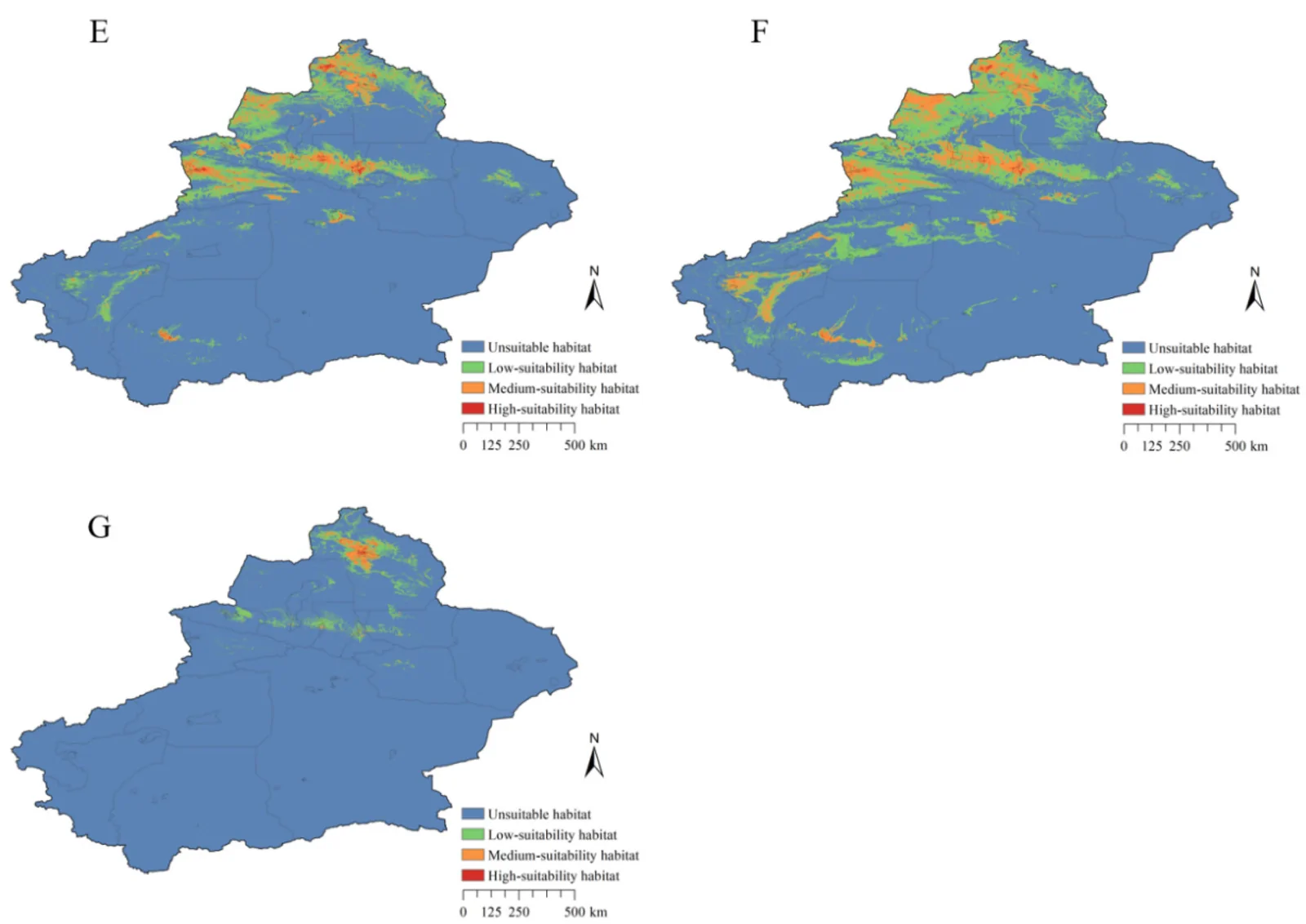
Distribution of suitable habitats of falconid birds in Xinjiang. (**A**) *F. cherrug*; (**B**) *F. columbarius*; (**C**) *F. naumanni*; (**D**) *F. peregrinus*; (**E**) *F. subbuteo*; (**F**) *F. tinnunculus*; (**G**) *F. vespertinus*.

The modeled current distributions align closely with Xinjiang’s topographic template of “three mountain ranges interspersed with two basins.” Medium-high suitability was concentrated along river corridors and piedmonts, including the southern slopes of the Altai and central-western Tianshan and their piedmonts, the Ili River Valley, and the northern Kunlun foothills. The interior basins (Junggar and Tarim) were predominantly low or unsuitable (Figure 3). Except for *F. vespertinus*, all species showed suitable areas in the central-western Tianshan, Altai, western Junggar mountain valley zones, and Kunlun northern foothills regions.

Two broad distributional patterns emerged: a Northern Xinjiang-biased distribution (e.g., *F. vespertinus*, *F. naumanni*), with a core high-suitability in the Irtysh and Ili basins and limited southern presence (a “core area” refers to a region where high-suitability habitats are spatially concentrated). Widespread northern and southern distributions (e.g., *F. cherrug*, *F. columbarius*, *F. peregrinus*), with *F. cherrug* showing the largest and most heterogeneous high-suitability network.

Quantitatively, the total suitable area (low + medium + high; 104 km^2^) varied among species: *F. cherrug* (38.14 × 104 km^2^; 22.9% of Xinjiang), *F. tinnunculus* (35.86 × 104 km^2^; 21.5%), *F. columbarius* (29.64 × 104 km^2^; 17.8%), *F. peregrinus* (24.10 × 104 km^2^; 14.5%), *F. naumanni* (23.69 × 104 km^2^; 14.2%), *F. subbuteo* (21.85 × 104 km^2^; 13.1%), and *F. vespertinus* (4.65 × 104 km^2^; 2.8%). Across species, unsuitable areas were dominant, followed by low-suitability areas. High-suitability habitats constituted the smallest fraction, with *F. cherrug* exhibiting the largest high-suitability extent (1.19 × 104 km^2^) and *F. vespertinus* the smallest (0.13 × 104 km^2^) (Table 8).

### 3.4. Current Relative Habitat Quality and Degradation Patterns

**Figure 4:**
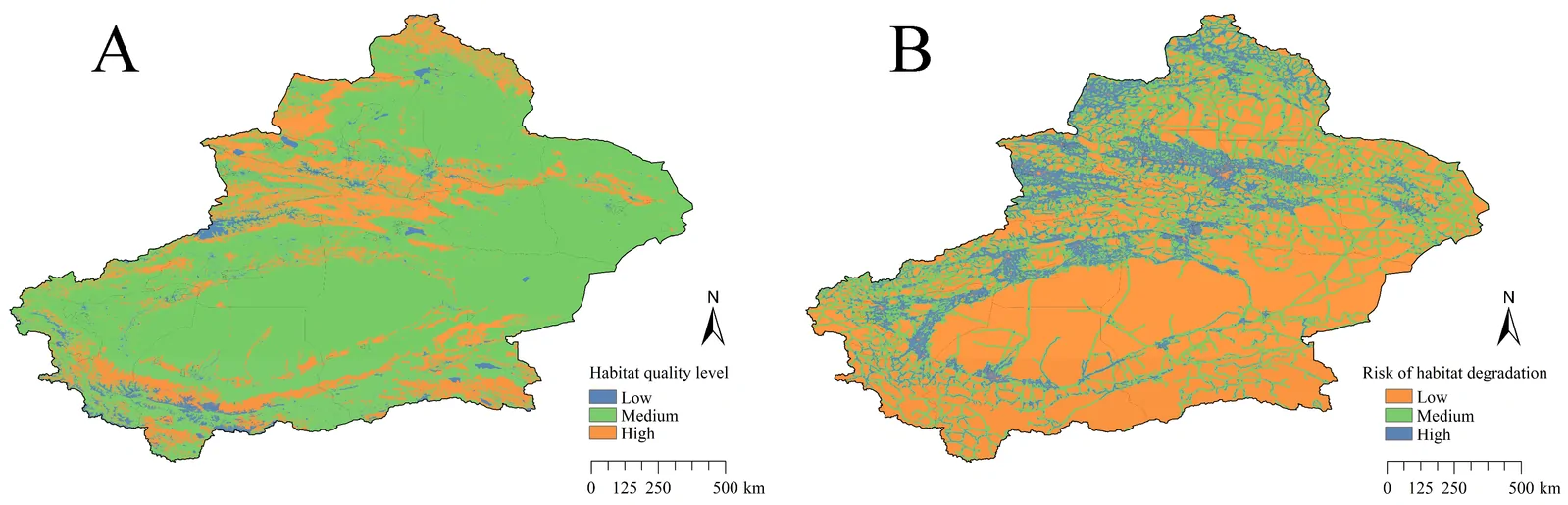
Relative habitat quality and habitat degradation risk in Xinjiang estimated using the InVEST Habitat Quality model: (**A**) relative habitat quality; (**B**) habitat degradation risk.

The InVEST Habitat Quality model showed that the mean relative habitat quality index in the Xinjiang Uygur Autonomous Region was 0.514, with a standard deviation of 0.185, indicating an overall moderate level of habitat quality and clear spatial heterogeneity across the study area. Based on the natural breaks classification method, the relative habitat quality index was divided into three classes: low, medium, and high. Medium-quality areas dominated the study region, followed by high-quality areas, whereas low-quality areas accounted for the smallest proportion (Figure 4A; Table 9). Spatially, areas with high relative habitat quality were mainly distributed in the piedmont zones of the Altai Mountains, the central-western Tianshan Mountains, the Ili River Valley, the Turpan-Hami Basin, the Kunlun Mountains, and the peripheral areas surrounding the Tarim Basin. High-quality areas in mountain foothills and river valleys were relatively continuous, whereas those around basin margins and some open landscapes were more patchily distributed. Areas with low relative habitat quality were mainly found in high-elevation mountain and snow-covered regions south of the Kunlun Mountains and in the central-western Tianshan Mountains. Low-quality habitats were also observed within several large water bodies, such as Sayram Lake, Ayding Lake, Ulungur Lake, and Bosten Lake.

The relative habitat degradation risk index was also classified into low, medium, and high levels using the natural breaks method. Low-risk areas accounted for the largest proportion, followed by medium-risk areas, whereas high-risk areas occupied a relatively smaller proportion (Figure 4B; Table 10). Compared with the spatial pattern of relative habitat quality, the distribution of relative degradation risk was more dispersed and showed a clear association with the transportation network. Medium-risk areas were mainly distributed in linear or network-like patterns along roads and railways, whereas high-risk areas were mostly clustered as patches around linear medium-risk zones. The spatial pattern of relative degradation risk was closely associated with transportation corridors. Medium-risk areas formed linear or network-like bands along roads and railways, whereas high-risk areas were mainly distributed as patches adjacent to these linear zones. High-degradation areas also occurred within or near some high-quality habitats.

A shows the spatial distribution of relative habitat quality, and B shows the spatial distribution of relative habitat degradation risk. The classified levels represent relative land-cover-based habitat conditions and degradation risk, rather than absolute ecological thresholds.

### 3.5. Suitability-Quality Mismatch and Protected-Area Representation

**Figure 5:**
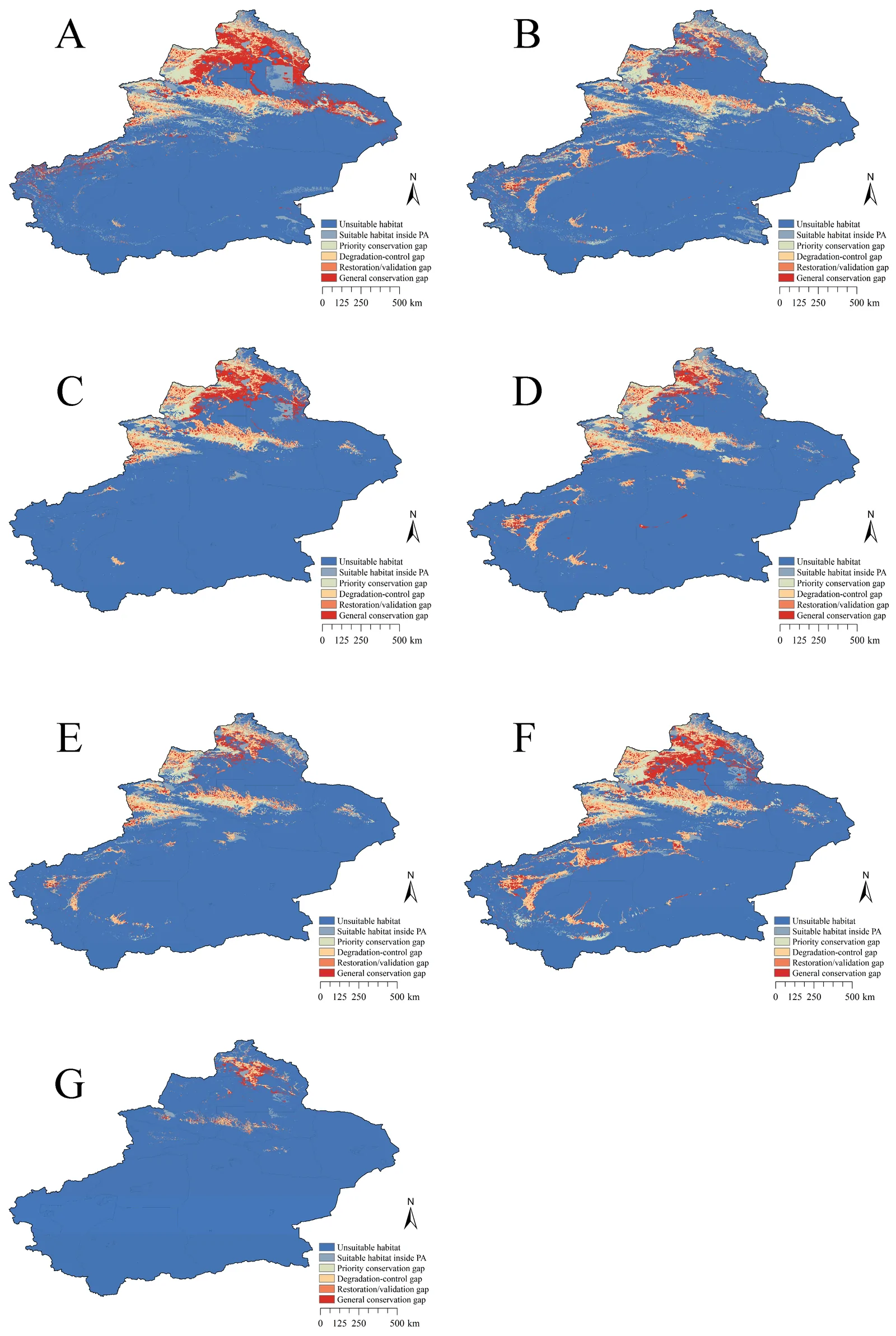
Spatial distribution of mutually exclusive conservation-gap categories for Falconidae species in Xinjiang. (**A**) *F. cherrug*; (**B**) *F. columbarius*; (**C**) *F. naumanni*; (**D**) *F. peregrinus*; (**E**) *F. subbuteo*; (**F**) *F. tinnunculus*; (**G**) *F. vespertinus*.

Overlay analyses further revealed extensive conservation gaps between predicted suitable habitats and the current protected-area network for the seven Falconidae species in Xinjiang (Figure 5; Table 11). The largest areas of suitable habitat outside protected areas were identified for *F. tinnunculus* and *F. cherrug* (304,866 and 303,205 km^2^, respectively), followed by *F. columbarius* (249,156 km^2^), *F. peregrinus* (204,335 km^2^), *F. naumanni* (193,059 km^2^), *F. subbuteo* (176,976 km^2^) and *F. vespertinus* (35,346 km^2^).

High-quality priority conservation gaps were most extensive for *F. cherrug*, *F. columbarius* and *F. tinnunculus*, covering 102,489, 97,490 and 94,413 km^2^, respectively. Substantial high-quality gaps were also found for *F. peregrinus*, *F. naumanni* and *F. subbuteo* (69,500–61,505 km^2^), whereas *F. vespertinus* showed a much smaller high-quality gap of 2752 km^2^. High-degradation management gaps were widespread, especially for *F. tinnunculus, F. columbarius*, *F. cherrug*, *F. peregrinus* and *F. subbuteo*, with areas ranging from 86,488 to 133,895 km^2^. *F. naumanni* also showed a large high-degradation gap of 80,273 km^2^, while *F. vespertinus* had a smaller gap of 16,312 km^2^.

High-quality and high-degradation areas also overlapped within unprotected suitable habitats for all species. These high-value but threatened gaps were largest for *F. tinnunculus*, *F. cherrug* and *F. columbarius* (36,647, 35,108 and 33,762 km^2^, respectively), followed by *F. naumanni*, *F. peregrinus* and *F. subbuteo* (23,940–25,630 km^2^). *F. vespertinus* showed the smallest overlap area, at 1716 km^2^.

Values are expressed in 10^4^ km^2^. Inside PA and Outside PA indicate suitable habitat inside and outside protected areas, respectively; suitable habitat outside protected areas was defined as the total conservation gap. Priority gap, low-quality management gap, high-degradation management gap and high-quality × high-degradation gap indicate unprotected suitable habitats with high habitat quality, low habitat quality, high degradation risk, and both high habitat quality and high degradation risk, respectively. General gap indicates other unprotected suitable habitats. These gap types were calculated as non-mutually exclusive categories and therefore do not necessarily sum to the total conservation gap area. PA is protected area.

Categories were derived from the overlay of predicted suitable habitats, protected-area coverage, relative habitat quality and degradation risk. Priority conservation gaps indicate unprotected suitable habitats with high habitat quality, degradation-control gaps indicate unprotected suitable habitats with high degradation risk, restoration/validation gaps indicate unprotected suitable habitats with low habitat quality, and general conservation gaps indicate other unprotected suitable habitats. PA is protected area.

### 3.6. Projected Future Fluctuations in Habitat Area

**Figure 6:**
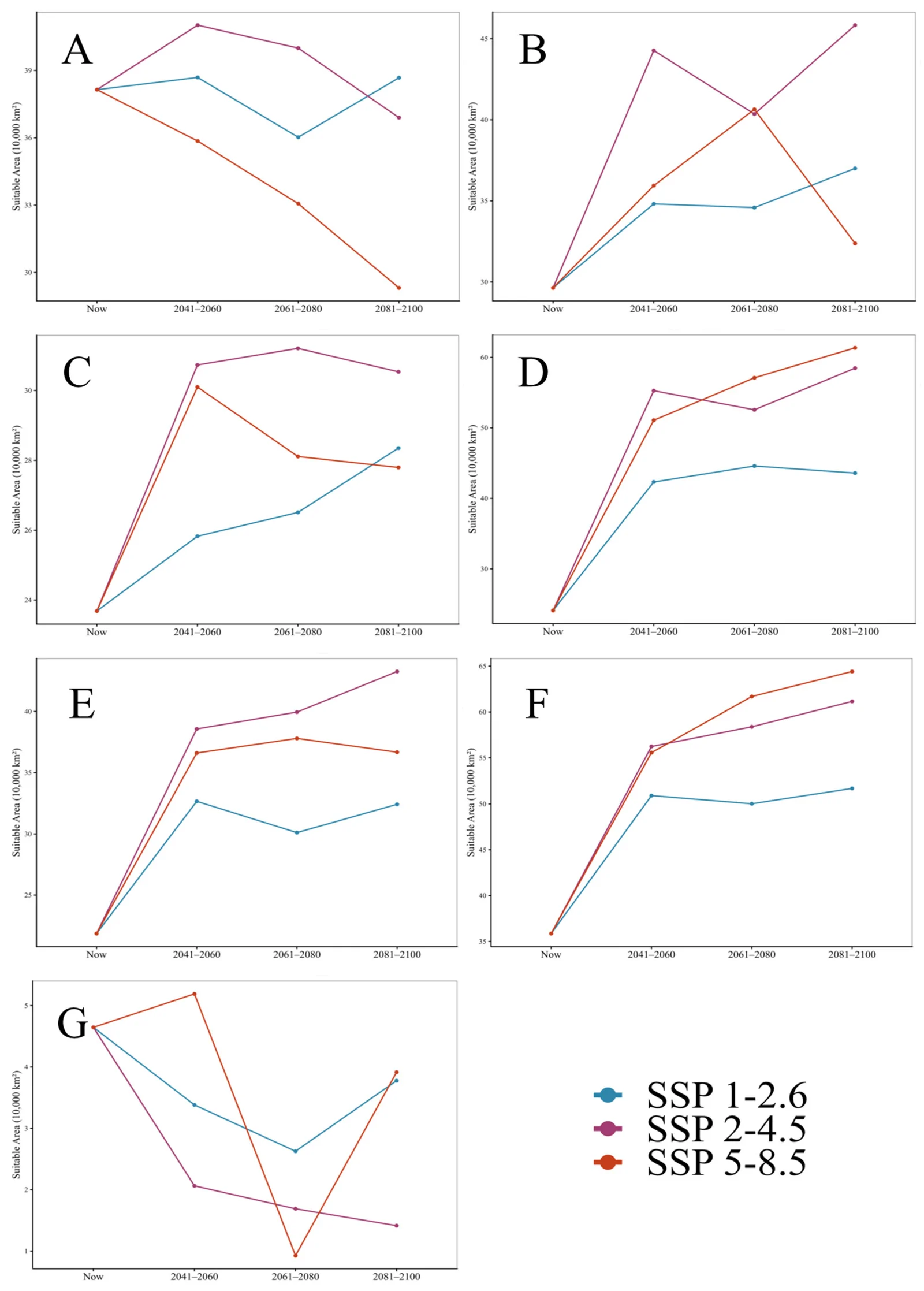
Future fluctuations in the suitable habitat area of falconid birds. (**A**) *F. cherrug*; (**B**) *F. columbarius*; (**C**) *F. naumanni*; (**D**) *F. peregrinus*; (**E**) *F. subbuteo*; (**F**) *F. tinnunculus*; (**G**) *F. vespertinus*.

Projected responses to future climate scenarios were strongly species- and scenario-dependent (Figure 6). Three main response types were identified in this study.

Expansion: *F. tinnunculus*, *F. peregrinus*, and *F. subbuteo* showed net increases in the total suitable area across all scenarios. Under SSP5-8.5 (2081–2100), *F. peregrinus* expanded to 61.35 × 104 km^2^ (+154.6%) and *F. tinnunculus* to 64.42 × 104 km^2^ (+79.5%) relative to current levels. Both also exhibited substantial increases in the high-suitability area (e.g., *F. tinnunculus* high-suitability +621% under SSP5-8.5).

Fluctuation/variable response: *F. columbarius* and *F. naumanni* showed moderate scenario-dependent changes in abundance. *F. columbarius* peaked under SSP2-4.5 (45.83 × 104 km^2^; +54.7%) but declined under late SSP5-8.5. *F. naumanni* experienced limited expansion (maximum +30.5%) with negligible gains in the core high-suitability area.

Declines: *F. vespertinus*, and under high emissions, *F. cherrug* exhibited a strong decline. *F. vespertinus* declined by 81.1% under SSP5-8.5 (to 0.88 × 104 km^2^ by 2081–2100), with a near loss of highly suitable habitat. *F. cherrug* showed progressive contraction under SSP5-8.5 (total suitable area decreasing over time).

Scenario effects were pronounced: the high-emission SSP5-8.5 scenario amplified habitat gains in species showing projected expansion and habitat losses in species showing projected contraction, whereas SSP2-4.5 produced comparatively moderate, more stable outcomes for several species. SSP1-2.6 produced the least change but did not fully reverse the decline in the most vulnerable species.

### 3.7. Barycenter Migration Trends

**Figure 7:**
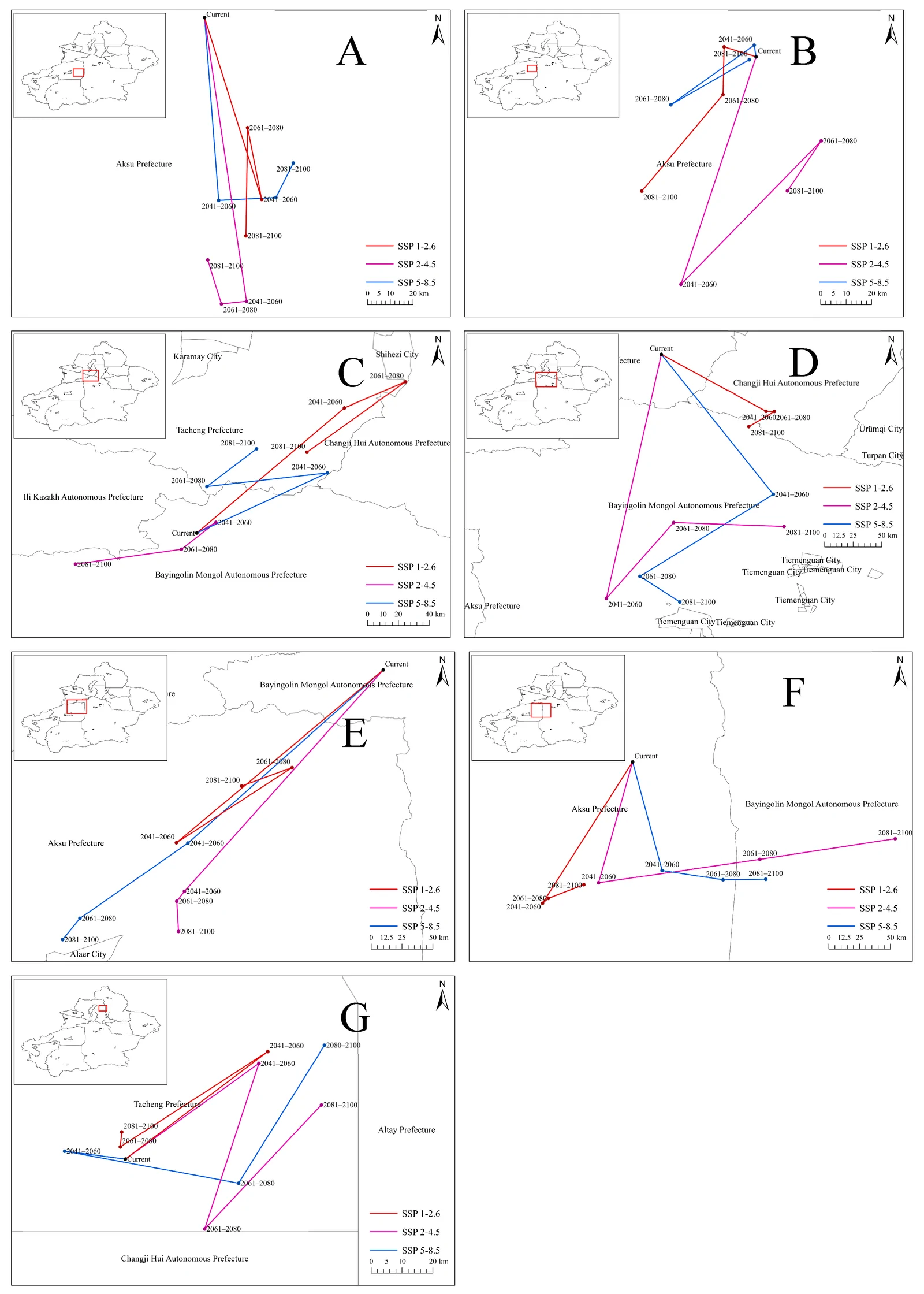
Future barycenter migration of suitable habitats of falconid birds. (**A**) *F. cherrug*; (**B**) *F. columbarius*; (**C**) *F. naumanni*; (**D**) *F. peregrinus*; (**E**) *F. subbuteo*; (**F**) *F. tinnunculus*; (**G**) *F. vespertinus*.

Across future climate scenarios (SSP1-2.6, SSP2-4.5, and SSP5-8.5) and periods (2041–2060, 2061–2080, and 2081–2100), the barycenters of suitable habitats for all seven falconid species shifted markedly; however, the direction, magnitude, and elevational response of these shifts differed substantially among species and scenarios (Figure 7). Among them, Falco subbuteo showed the strongest directional consistency, with barycenters shifting southwestward (211–223°) in all scenarios and time periods. The shift distance increased with emission intensity and time, reaching 291.89 km under SSP5-8.5 in 2081–2100, whereas centroid elevation declined continuously by 767–1458 m, indicating an increasing tendency toward lower-elevation southwestern areas. In contrast, *F. peregrinus* showed the strongest scenario dependence. Under SSP1-2.6, its barycenter shifted steadily southeastward (83–88 km) with substantial elevational increases (+954 to +1794 m), whereas under SSP2-4.5 and SSP5-8.5, both horizontal and vertical responses became more complex, including long-distance southward shifts and stage-dependent reversals in elevation trends. Under SSP5-8.5, centroid elevation increased during 2041–2080 (+1056 to +1097 m) but declined in 2081–2100 (−474 m), accompanied by a pronounced southward shift of 216.90 km.

Compared with these strongly shifting species, *F. tinnunculus* was characterized mainly by medium-distance horizontal redistribution (90–172 km), with the migration direction gradually changing from southward to southeastward or eastward in later periods and only weak elevational variation. In contrast, *F. vespertinus* showed the shortest barycenter displacement, with shifts of less than 60 km in most periods, variable directions among the northeast, north, and east, and only minor elevational changes. *F. cherrug* and *F. columbarius* also exhibited relatively limited spatial adjustments, with horizontal shifts of 50–125 km and 2–92 km, respectively, and only slight elevational fluctuations. *F. naumanni* differed from the other species by predominantly combining northeastward shifts of moderate distance with strong, stage-dependent elevational declines. Under SSP1-2.6, its barycenter moved continuously northeastward (74–139 km) while elevation dropped sharply by 656–2867 m; under SSP2-4.5, shift magnitude decreased and direction turned southwestward or westward, while elevation continued to decline (−533 to −848 m); under SSP5-8.5, northeastward movement again predominated and was generally accompanied by elevation loss (−317 to −1124 m), except for a temporary northward shift and slight elevational increase (+239 m) during 2061–2080. For several species, barycenter displacement increased under higher-emission scenarios and in later periods.

## 4. Discussion

### 4.1. Main Environmental Drives of Falconid Habitat Suitability

LUCC emerged as a dominant predictor for six species, indicating a close association between falconid habitat suitability and landscape composition in Xinjiang. Artificial surfaces, cropland, wetlands, and water-associated landscapes may provide prey resources, foraging opportunities, perching structures, or nesting substrates for some falconid species [91,92,93,94]. However, the comparatively high predicted suitability associated with artificial surfaces should be interpreted cautiously. Occurrence records may be concentrated near roads, settlements, and other accessible locations, and spatial thinning reduces local clustering but does not fully account for uneven observation effort. Therefore, the observed LUCC associations should be treated as ecological hypotheses requiring validation through target-group background sampling, bias surfaces, independent field surveys, or other explicit sampling-bias correction approaches. The threshold-like increase in suitability above an NDVI value of approximately 0.2 suggests that a minimum level of vegetation cover may be required to sustain prey availability and stable falconid activity [95,96]. The subsequent decline at high NDVI values for several species may reflect reduced hunting efficiency in dense vegetation, whereas the different response of *F. naumanni* may be associated with broader use of productive grassland or agricultural mosaic habitats. These contrasting response shapes emphasize that vegetation cover affects falconid species through species-specific foraging and habitat-use strategies rather than through a uniform positive relationship.

The species-specific responses to BIO19 and BIO15 indicate differences in tolerance to winter moisture availability and precipitation seasonality [97,98]. Moderate winter precipitation may improve vegetation conditions and prey availability, whereas excessive precipitation, rainfall, or snow cover may reduce prey accessibility and hunting efficiency. The strong decline in the predicted suitability of *F. vespertinus* under increasing precipitation seasonality suggests that this species may be particularly sensitive to unstable moisture regimes.

Taken together, the contributions of LUCC, NDVI, and precipitation variables indicate that falconid distributions in Xinjiang are jointly constrained by climatic conditions and landscape structure rather than by climate alone. This result supports the inclusion of both climatic and non-climatic predictors when modeling raptor distributions in arid and semi-arid landscapes.

### 4.2. Spatial Patterns and Future Fluctuations in Suitable Habitat

Current suitable habitats display a pronounced “mountain-valley” aggregation: medium-high suitability is concentrated along river corridors on the southern slope of the Altai Mountains, central-western Tianshan Mountains, and northern Kunlun foothills, whereas low suitability dominates the Junggar and Tarim basin interiors. This pattern reflects the hydrothermal gradients imposed by Xinjiang’s “three mountains with two basins” topography: elevational changes create spatial heterogeneity in temperature and precipitation, which drive vegetation patterns and prey distributions, producing more favorable conditions in piedmont oases and river valleys. These findings support the prioritization of conservation efforts in piedmont and riverine corridors, particularly the Altai-Northern Tianshan interface and the Kunlun foothills, where core habitat and connectivity are most critical [99,100,101].

Future projections reveal divergent and species-specific trajectories for these species. Under most scenarios, 60% of the modeled species (e.g., *F. tinnunculus* and *F. peregrinus*) showed net habitat expansion, while others (notably *F. vespertinus* and *F. cherrug*) were projected to contract, with *F. vespertinus* being particularly vulnerable under high-emission scenarios (SSP5-8.5). These contrasting responses likely reflect differences in ecological tolerance, life history, and habitat specialization. Some falconids appear capable of exploiting newly favorable areas under warming and humidification, whereas others are confined to narrower climatic envelopes or habitat dependencies. Scenario sensitivity was evident: SSP2-4.5 produced relatively moderate changes for most species, whereas SSP5-8.5 amplified losses for the sensitive taxa. Consequently, contraction-prone species (e.g., *F. vespertinus*) and their core areas (e.g., the Irtysh River Basin) should be prioritized for targeted protection, and adaptive management should be tailored to species-specific physiology and ecology [102].

### 4.3. Conservation Gaps and Climate-Adaptive Conservation Priorities

The combined MaxEnt-InVEST-protected-area overlay revealed clear conservation gaps for Falconidae species in Xinjiang. Large areas of predicted suitable habitat occurred outside the current protected-area network, especially for *F. tinnunculus*, *F. cherrug*, *F. columbarius* and *F. peregrinus*. These findings demonstrate that the existing protected-area network does not fully represent the suitable habitats of Falconidae in Xinjiang, particularly where unprotected suitable habitats coincide with high habitat quality or elevated degradation risk. Conservation planning should therefore distinguish among priority protection gaps, degradation-control gaps, and restoration or field-validation gaps rather than treating all unprotected suitable habitats as equivalent. Such a gap-analysis perspective is central to systematic conservation planning, which aims to assess the representativeness of existing protected areas and identify additional areas needed to meet conservation objectives [37,38].

Unprotected suitable habitats with high relative habitat quality represent priority conservation gaps. These areas were most extensive for *F. cherrug*, *F. columbarius* and *F. tinnunculus*, indicating that they should be prioritized for protected-area expansion, ECRs (ecological conservation redlines, which are designated areas in China with critical ecological functions or high ecological sensitivity, established to safeguard ecological security and biodiversity), other effective area-based conservation measures or targeted habitat management. In contrast, unprotected suitable habitats with high degradation risk should be treated as degradation-control gaps, where disturbance reduction, infrastructure regulation and habitat management are needed. This interpretation is consistent with the logic of the InVEST Habitat Quality model, which evaluates habitat condition by combining land-use/land-cover information with threat sources and habitat sensitivity [103]. Areas with low relative habitat quality should be interpreted cautiously as restoration or validation gaps, because they may represent either genuinely degraded habitats or open arid landscapes still used by falcons for foraging. Field validation is therefore important when model outputs are used to support conservation decisions [104].

These results also show that high MaxEnt suitability does not necessarily indicate high habitat quality. MaxEnt reflects environmental suitability based on climatic and environmental conditions associated with species occurrences, whereas InVEST estimates relative habitat quality from land cover, threat sources and habitat sensitivity [28,29]. Therefore, climatically suitable areas may still have low habitat quality or high degradation risk under strong human disturbance. Integrating both approaches helps distinguish environmental suitability, land-cover-based habitat condition and protection status, thereby reducing the risk of interpreting habitat suitability maps as direct measures of habitat quality [105,106]. The spatial juxtaposition of high relative habitat quality and elevated degradation risk, particularly along transportation corridors, further demonstrates that habitat condition and anthropogenic pressure are not spatially equivalent. Landscapes with favorable land-cover-based habitat conditions may still be exposed to substantial road, construction, or agricultural disturbance. This mismatch supports the joint use of habitat suitability, habitat quality, degradation risk, and protected-area coverage when identifying conservation priorities. The identified gaps are also relevant for climate-adaptive conservation. Because Falconidae species showed different projected habitat shifts, conservation strategies should be species-specific. Species with extensive current gaps and large high-quality unprotected habitats, such as F. cherrug, *F. tinnunculus* and *F. columbarius*, require immediate protection of current priority habitats and maintenance of future movement pathways. Species whose suitable habitats overlap strongly with degradation risk, such as *F. peregrinus* and *F. subbuteo*, require combined climate-refugia protection and disturbance management. For range-restricted species such as *F. vespertinus*, even small conservation gaps may increase vulnerability to fragmentation and future climate change. These strategies are consistent with climate-integrated conservation approaches that emphasize protected-area adjustment, connectivity maintenance and management of multiple threats under climate change [107,108].

### 4.4. Projected Barycenter Shifts and Conservation Implications

All seven species exhibited significant barycenter shifts under future scenarios; however, the directions, distances, and elevational responses varied among species and scenarios. Five species (*F. subbuteo*, *F. peregrinus*, *F. tinnunculus*, *F. cherrug*, *F. columbarius*) generally shifted southwestward or southward in many scenarios, whereas *F. vespertinus* and *F. naumanni* tended to shift northeastward or showed directional variability. Vertical responses were heterogeneous, with *F. subbuteo*, *F. peregrinus*, and *F. naumanni* showing pronounced elevational adjustments, although not uniformly upward or downward. These patterns align with the general expectations of poleward/upslope redistributions under warming but emphasize that responses are context- and species-dependent [109,110]. The limited elevational variation in *F. tinnunculus* suggests that its projected redistribution may be driven mainly by horizontal climatic gradients. By contrast, the small barycenter displacement of *F. vespertinus*, despite substantial habitat contraction, may reflect localized redistribution and increasing fragmentation rather than effective tracking of shifting climatic conditions. The greater displacement of several species under stronger emission scenarios further indicates that future habitat reorganization may extend beyond the capacity of static protected-area boundaries.

*F. subbuteo* displayed the most consistent southwestward and downslope shifts (elevational changes of −767 to −1458 m). This pattern likely reflects its strong sensitivity to temperature seasonality (BIO3:12.9% contribution) and warmer quarter temperatures (BIO10:3.5% contribution) (Table 5), suggesting a preference shift toward lower-elevation, warmer/humidifying habitats as these conditions expand or become more favorable in those areas. *F. peregrinus* showed more complex dynamics, including a reversal from an elevational increase to a decline under late SSP5-8.5, indicating nonlinear responses to intensifying climate stressors. Species with limited barycenter movement (e.g., *F. vespertinus* < 60 km in most periods) are likely to experience local fragmentation and range contraction, increasing the extinction risk owing to isolation. Under the SSP5-8.5 (high-emission) scenario, climate-induced changes in temperature and precipitation may, over time, exceed the survival tolerance thresholds of some species, thereby exerting significant pressure on their persistence and resulting in a substantial loss of suitable habitat. [111,112,113].

Migration distances increased with emission intensity and time, with maximum shifts (e.g., *F. subbuteo* ~292 km) that, while within the dispersal capacity of many raptors, may be constrained by landscape connectivity, barriers, and availability of stepping-stone habitats. Conservation planning should therefore be dynamic and species-specific: establish cross-regional ecological corridors for long-distance shifting species (*F. subbuteo* and *F. naumanni*), particularly focusing on connecting piedmont oases and river valleys in the central-western Tianshan and northern Kunlun foothills, which serve as crucial current and projected future habitat clusters. For fragmentation-prone species such as *F. vespertinus*, which faces substantial declines and localized shifts, strengthening the protection and habitat quality of the remaining core patches, particularly in the Irtysh River Basin, where its current high-suitability areas are concentrated (Figure 3G) and are projected to face significant contraction, is necessary, as is evaluating the protection value across elevational gradients for species with complex vertical responses (*F. peregrinus*). Maintaining and restoring habitat connectivity, particularly along predicted migration trajectories and riverine corridors, is essential for facilitating climate-driven range adjustments [8].

### 4.5. Limitations and Future Research

Several uncertainties should be considered when interpreting the results. First, future projections were based on a single global climate model, BCC-CSM2-MR, and a single future LUCC dataset. The ten MaxEnt replicates quantified internal model variability but did not represent uncertainty among alternative climate models or land-use projections. Second, MaxEnt outputs may have been affected by uneven occurrence-record sampling, predictor selection, and threshold choice. Spatial thinning reduced local clustering but did not fully account for spatial variation in observation effort. Third, the InVEST Habitat Quality model provides a relative land-cover-based index and does not explicitly represent prey availability, nesting-site availability, species-specific disturbance tolerance, or fine-scale habitat conditions [41,114]. Finally, protected-area overlap measures spatial representation rather than management effectiveness. Future studies should incorporate multi-model climate ensembles, alternative LUCC projections, explicit sampling-bias correction, connectivity and demographic models, and independent field or telemetry data to quantify uncertainty and validate the projected habitat changes.

## 5. Conclusions

Using 2731 occurrence records, 26 environmental predictors, and an optimized MaxEnt framework, we assessed current and future habitat suitability for seven falconid species in Xinjiang. Suitable habitats were mainly concentrated in piedmont oases, river valleys, and mountain foothills. Future responses were strongly species-specific: *Falco tinnunculus*, *F. peregrinus*, and *F. subbuteo* generally showed habitat expansion or persistence, whereas *F. vespertinus* and *F. cherrug* were projected to decline, particularly under high-emission scenarios. All species exhibited shifts in suitable-habitat barycenters, although the direction, distance, and elevational response varied among species and scenarios.

Integrating MaxEnt predictions with InVEST-based habitat quality, degradation risk, and protected-area overlays showed that environmental suitability did not necessarily correspond to favorable habitat condition or adequate protection. Extensive suitable habitats occurred outside protected areas, including high-quality priority gaps and areas exposed to substantial degradation risk. Conservation planning should therefore adopt species-specific and climate-adaptive strategies that protect high-quality unprotected habitats, reduce disturbance in threatened areas, restore or validate low-quality suitable habitats, and maintain landscape connectivity. Future studies should incorporate multiple climate and land-use projections, connectivity and demographic models, and independent movement or monitoring data to improve projection robustness.

## Figures and Tables

**Table 1:** Environmental variables included in this study.

Data Type	Environmental Factor	Code	Unit
Climatic Factor	Annual mean temperature	BIO1	°C
	Mean diurnal range	BIO2	°C
	Isothermality	BIO3	%
	Temperature seasonality	BIO4	-
	Max temperature of the warmest month	BIO5	°C
	Min temperature of the coldest month	BIO6	°C
	Temperature annual range	BIO7	°C
	Mean temperature of the wettest quarter	BIO8	°C
	Mean temperature of the driest quarter	BIO9	°C
	Mean temperature of the warmest quarter	BIO10	°C
	Mean temperature of the coldest quarter	BIO11	°C
	Annual precipitation	BIO12	mm
	Precipitation of the wettest month	BIO13	mm
	Precipitation of the driest month	BIO14	mm
	Precipitation seasonality	BIO15	-
	Precipitation of the wettest quarter	BIO16	mm
	Precipitation of the driest quarter	BIO17	mm
	Precipitation of the warmest quarter	BIO18	mm
	Precipitation of the coldest quarter	BIO19	mm
Topographic Factor	Elevation	ELE	m
	Aspect	ASPECT	-
	Slope	SLOPE	-
Natural Factor	Land use and land cover change	LUCC	-
	Distance to water system	Dis_ws	m
	Distance to the nature reserve	Dis_nr	
	Normalized difference vegetation index	NDVI	-

**Table 2:** Threat factor parameters.

Threat	Max_Dist/km	Weight	Decay
roads	5	0.7	linear
builtupland	8	0.8	exponential
cropland	3	0.5	linear
bareland	4	0.4	linear

**Table 3:** Habitats suitability of different land use types and sensitivities.

Land Use Type	Habitat Suitability	Roads	Builtupland	Cropland	Bareland
cropland	0.5	0.4	0.4	0.2	0.2
forest	0.5	0.7	0.7	0.5	0.4
grassland	0.9	0.7	0.8	0.5	0.4
shrubland	0.65	0.7	0.7	0.5	0.4
wetland	0.6	0.6	0.6	0.5	0.3
water	0.2	0.3	0.3	0.2	0.2
builtupland	0	0	0	0	0
bareland	0.45	0.5	0.5	0.2	0.1
snow_ice	0.05	0.1	0.1	0	0.1

**Table 4:** Protection status and numbers of valid occurrence records of Falconidae species in Xinjiang.

English Name	Scientific Name	IUCN Red List Conservation Category	National Priority Conservation Category in China	Valid Occurrence Sites
Amur Falcon	*F. amurensis*	LC	II	2
Saker Falcon	*F. cherrug*	EN	I	163
Merlin	*F. columbarius*	LC	II	105
Lesser Kestrel	*F. naumanni*	LC	II	517
Peregrine Falcon	*F. peregrinus*	LC	II	114
Gyrfalcon	*F. rusticolus*	LC	I	3
Eurasian Hobby	*F. subbuteo*	LC	II	405
Common Kestrel	*F. tinnunculus*	LC	II	1327
Red-footed Falcon	*F. vespertinus*	VU	II	100

**Table 5:** Results of environmental variable screening for seven Falconidae species in Xinjiang.

Code	*F. cherrug*	*F. columbarius*	*F. naumanni*	*F. peregrinus*	*F. subbuteo*	*F. tinnunculus*	*F. verspertinus*
BIO1	●	○	●	●	○	●	○
BIO2	●	●	●	●	●	●	●
BIO3	●	●	●	●	●	●	●
BIO4	●	●	○	○	●	●	○
BIO5	○	○	○	○	○	○	○
BIO6	○	○	○	○	●	○	●
BIO7	○	○	●	●	○	○	○
BIO8	○	○	○	○	○	○	●
BIO9	○	●	○	○	○	○	○
BIO10	○	○	○	○	●	○	○
BIO11	○	○	○	○	○	○	○
BIO12	●	●	○	○	○	○	○
BIO13	○	○	○	○	○	○	●
BIO14	○	○	○	●	○	○	○
BIO15	●	●	●	●	●	●	●
BIO16	○	○	●	○	○	○	○
BIO17	○	○	○	○	○	○	●
BIO18	○	○	○	○	●	●	○
BIO19	●	●	●	○	●	●	○
Elevation	○	○	○	○	○	○	●
ASPECT	●	●	○	●	○	●	●
SLOPE	●	●	●	●	●	●	○
LUCC	●	●	●	●	●	●	●
Dis_ws	●	●	●	●	●	●	●
Dis_nr	●	●	●	●	●	●	●
NDVI	●	●	●	●	●	●	●

○ indicates that the variable was excluded from the MaxEnt model for the corresponding species. ● indicates that a variable was included in the MaxEnt model for the corresponding species.

**Table 6:** Enmeval optimization metrics for the MaxEnt model.

Species	Type	RM	FC	Delta.AICc	Avg.Diff.AUC	Mean.OR10%
*F. cherrug*	Default	1	LQHP	125.918	0.023	0.178
	Optimized	2.5	LQH	0.000	0.025	0.142
*F. columbarius*	Default	1	LQHP	163.924	0.041	0.259
	Optimized	0.5	LQ	0.000	0.029	0.153
*F. naumanni*	Default	1	LQHP	3.090	0.012	0.124
	Optimized	1	QHP	0.000	0.012	0.126
*F. peregrinus*	Default	1	LQHP	126.497	0.052	0.169
	Optimized	2	LQ	0.000	0.052	0.122
*F. subbuteo*	Default	1	LQHP	2.398	0.011	0.126
	Optimized	1.5	LQHPT	0.000	0.009	0.121
*F. tinnunculus*	Default	1	LQHP	0.000	0.012	0.106
	Optimized	-	-	-	-	-
*F. vespertinus*	Default	1	LQHP	95.958	0.013	0.190
	Optimized	0.5	LQ	0.000	0.010	0.160

AICc: Akaike information criterion corrected; Delta.AICc: AICc minimum information criterion AICc value; Avg.diff.AUC: Average difference between the training and testing AUC; Mean.OR10:10% test omission rate. The optimization metrics for *F. tinnunculus* were not applicable, as its default settings already yielded ΔAICc = 0, indicating optimal complexity.

**Table 7:** Percent contribution (%) of environmental variables for seven Falconidae species in Xinjiang.

Code	*F. cherrug*	*F. columbarius*	*F. naumanni*	*F. peregrinus*	*F. subbuteo*	*F. tinnunculus*	*F. vespertinus*
BIO1	5.7	○	4.3	2.9	○	7.0	○
BIO2	5.3	2.4	3.4	8.6	2.2	1.6	2.3
BIO3	0.4	3.9	3.3	7.7	12.9	2.3	10.4
BIO4	0.2	1.4	○	○	3.3	2.5	○
BIO5	○	○	○	○	○	○	○
BIO6	○	○	○	○	1.0	○	0.8
BIO7	○	○	3.4	1.8	○	○	○
BIO8	○	○	○	○	○	○	0.1
BIO9	○	1.6	○	○	○	○	○
BIO10	○	○	○	○	3.5	○	○
BIO11	○	○	○	○	○	○	○
BIO12	0.1	0.6	○	○	○	○	○
BIO13	○	○	○	○	○	○	1.3
BIO14	○	○	○	0.3	○	○	○
BIO15	1.2	4.3	1.4	10.7	9.2	4.5	36.6
BIO16	○	○	0.7	○	○	○	○
BIO17	○	○	○	○	○	○	3.0
BIO18	○	○	○	○	0.3	1.2	○
BIO19	36.7	0.1	55.4	○	6.6	18.3	○
Elevation	○	○	○	○	○	○	1.4
ASPECT	0.1	1.2	○	0.3	○	0.1	1.3
SLOPE	3.1	1.8	0.1	0.5	0.5	0.3	○
LUCC	18.1	76.1	14.3	64.9	20.4	37.0	29.4
Dis_ws	15.3	2.2	7.4	1.0	8.7	11.0	4.5
Dis_nr	2.3	1.5	1.2	0.4	1.6	1.1	7.4
NDVI	11.6	2.9	5.1	0.9	29.7	13.0	1.7

○ indicates that the variable was excluded from the MaxEnt model for the corresponding species; numbers indicate that the variable was included in the MaxEnt model for the corresponding species and represents its percentage contribution.

**Table 8:** Current suitable habitat area of falconid birds in Xinjiang (10^4^ km^2^).

Habitat Class	*F. cherrug*	*F. columbarius*	*F. naumanni*	*F. peregrinus*	*F. subbuteo*	*F. tinnunculus*	*F. vespertinus*
Proportion of un habitat	0.77089	0.82197	0.85772	0.85527	0.86873	0.78459	0.97210
Proportion of low habitat	0.15724	0.15275	0.10110	0.12346	0.09353	0.15456	0.02088
Proportion of medium habitat	0.06472	0.02097	0.03853	0.01674	0.03445	0.05890	0.00625
Proportion of high habitat	0.00715	0.00431	0.00264	0.00452	0.00329	0.00195	0.00078
Area of un habitat	128.34539	136.84921	142.80252	142.39448	144.63557	130.62613	161.84429
Area of low habitat	26.17836	25.43082	16.83182	20.55482	15.57259	25.73277	3.47605
Area of medium habitat	10.77598	3.49211	6.41556	2.78754	5.73479	9.80568	1.04056
Area of high habitat	1.19028	0.71786	0.44010	0.75315	0.54705	0.32542	0.12910
Total habitat area	38.14461	29.64079	23.68748	24.09552	21.85443	35.86387	4.64571

“un habitat” denotes unsuitable habitat; “low habitat” denotes low-suitability habitat; “medium habitat” denotes medium-suitability habitat; and “high habitat” denotes high-suitability habitat.

**Table 9:** Areas and percentages of relative habitat quality classes in Xinjiang (10^4^ km^2^).

Habitat Quality Levels	Area (10^4^ km^2^)	Percentage (%)
Low	5.87	3.53%
Medium	126.67	76.09%
High	33.95	20.38%

**Table 10:** Areas and percentages of relative habitat degradation risk classes in Xinjiang (10^4^ km^2^).

Habitat Degradation Severity Levels	Area (10^4^ km^2^)	Percentage (%)
Low	95.96	57.63%
Medium	49.32	29.63%
High	21.21	12.74%

**Table 11:** Areas of protected-area coverage and conservation-gap types for suitable habitats of falconid birds in Xinjiang (10^4^ km^2^).

Habitat Category	*F. cherrug*	*F. columbarius*	*F. naumanni*	*F. peregrinus*	*F. subbuteo*	*F. tinnunculus*	*F. vespertinus*
Inside PA	5.59356	3.49458	2.70985	2.31860	2.86470	3.75446	0.70075
Outside PA	30.32046	24.91561	19.30589	20.43346	17.69759	30.48660	3.53464
Priority gap	10.24886	9.74903	6.52155	6.95001	6.15054	9.44125	0.27523
Low-quality management gap	0.98716	1.25549	0.76551	1.16743	0.95585	1.30683	0.31621
High-degradation management gap	10.06422	11.55478	8.02726	9.65437	8.6488	13.38951	1.63118
High-quality × high-degradation gap	3.51084	3.37623	2.56303	2.53497	2.39404	3.66468	0.17163
General gap	12.55069	5.75563	6.56995	5.21736	4.3578	10.04063	1.48903

## Data Availability

The raw data supporting the conclusions of this article will be made available by the authors on request.

## Supplementary Materials

The following supporting information can be downloaded at: https://www.mdpi.com/article/10.3390/ani16172650/s1.
