# Supplementary material for: Habitat Shifts and Conservation Challenges of Falconidae Under Climate Change in Northwestern China
Source: Animals (Basel). 2026 Aug 24;16(17):2650. doi: 10.3390/ani16172650 (PMC13564317; doi:10.3390/ani16172650)
Supplement: Supplementary file 1 [file animals-16-02650-s001.zip › animals-4414281-supplementary.pdf]

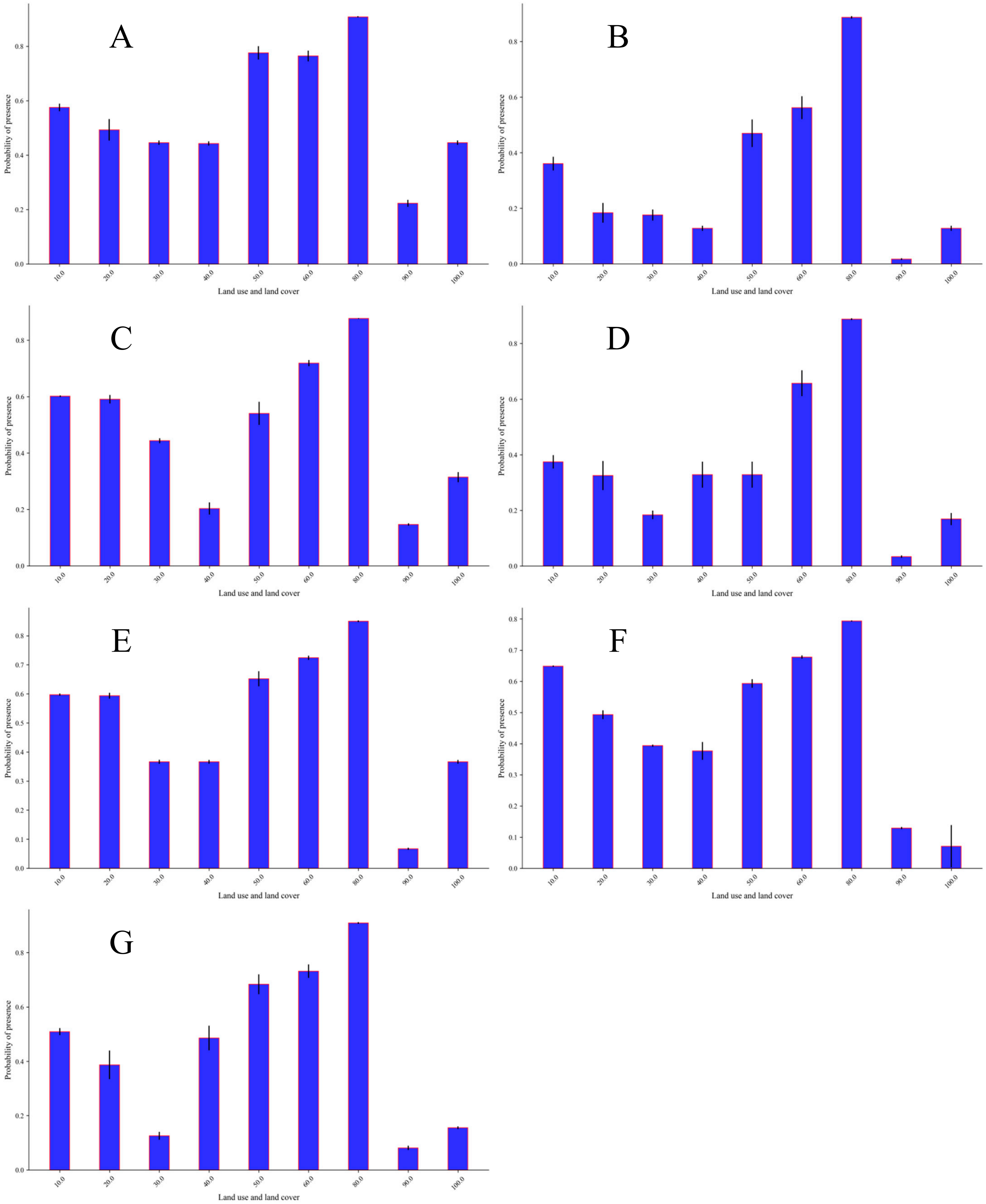

Supplementary Figure S1 Response curves of LUCC for different falcon species

A *F.cherrug*; B *F.columbarius*; C *F.naumanni*; D *F.peregrinus*; E *F.subbuteo*; F *F.tinnunculus*; G *F.vespertinus*

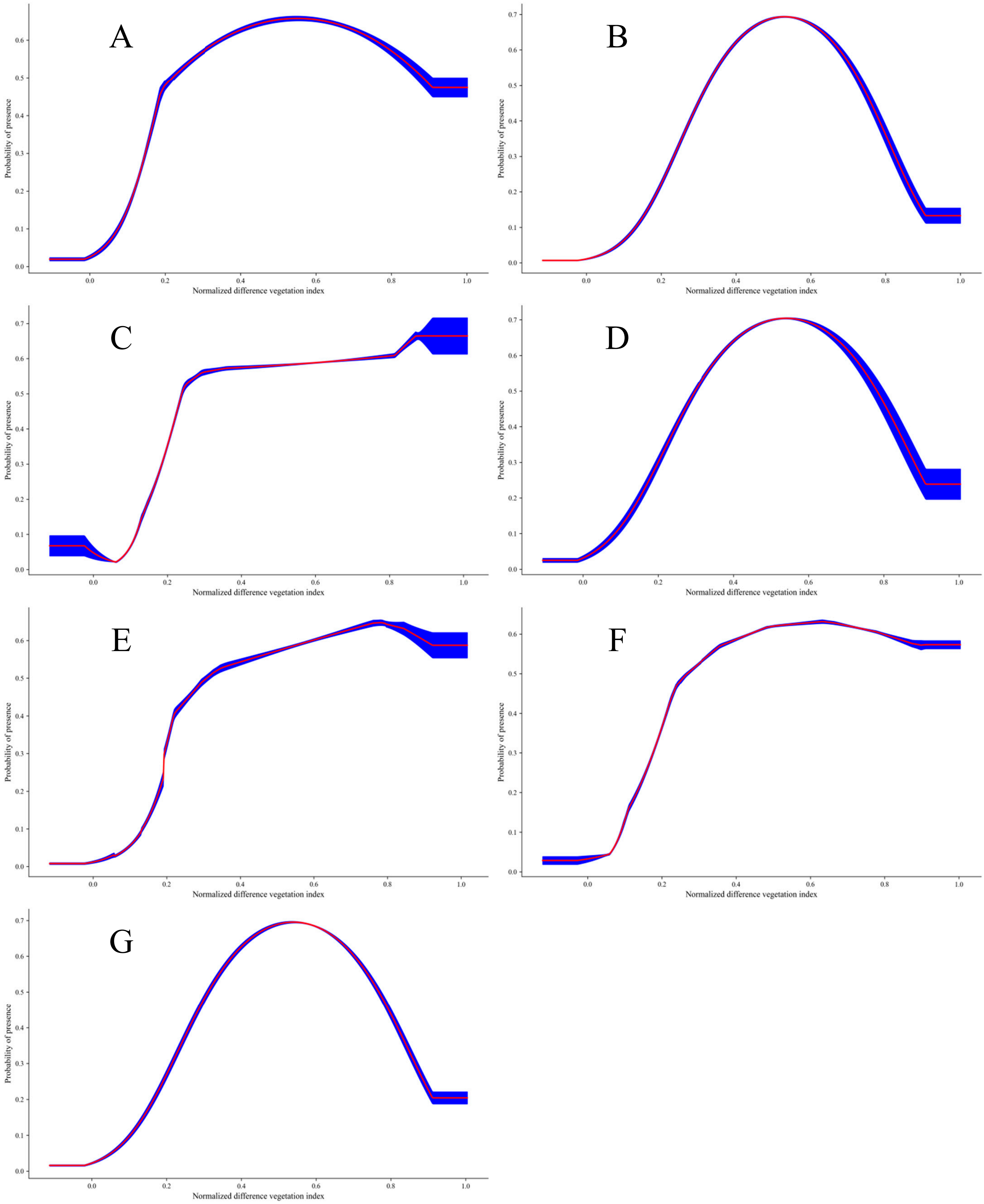

Supplementary Figure S2 Response curves of NDVI for different falcon species

A *F.cherrug*; B *F.columbarius*; C *F.naumanni*; D *F.peregrinus*; E *F.subbuteo*; F *F.tinnunculus*; G *F.vespertinus*

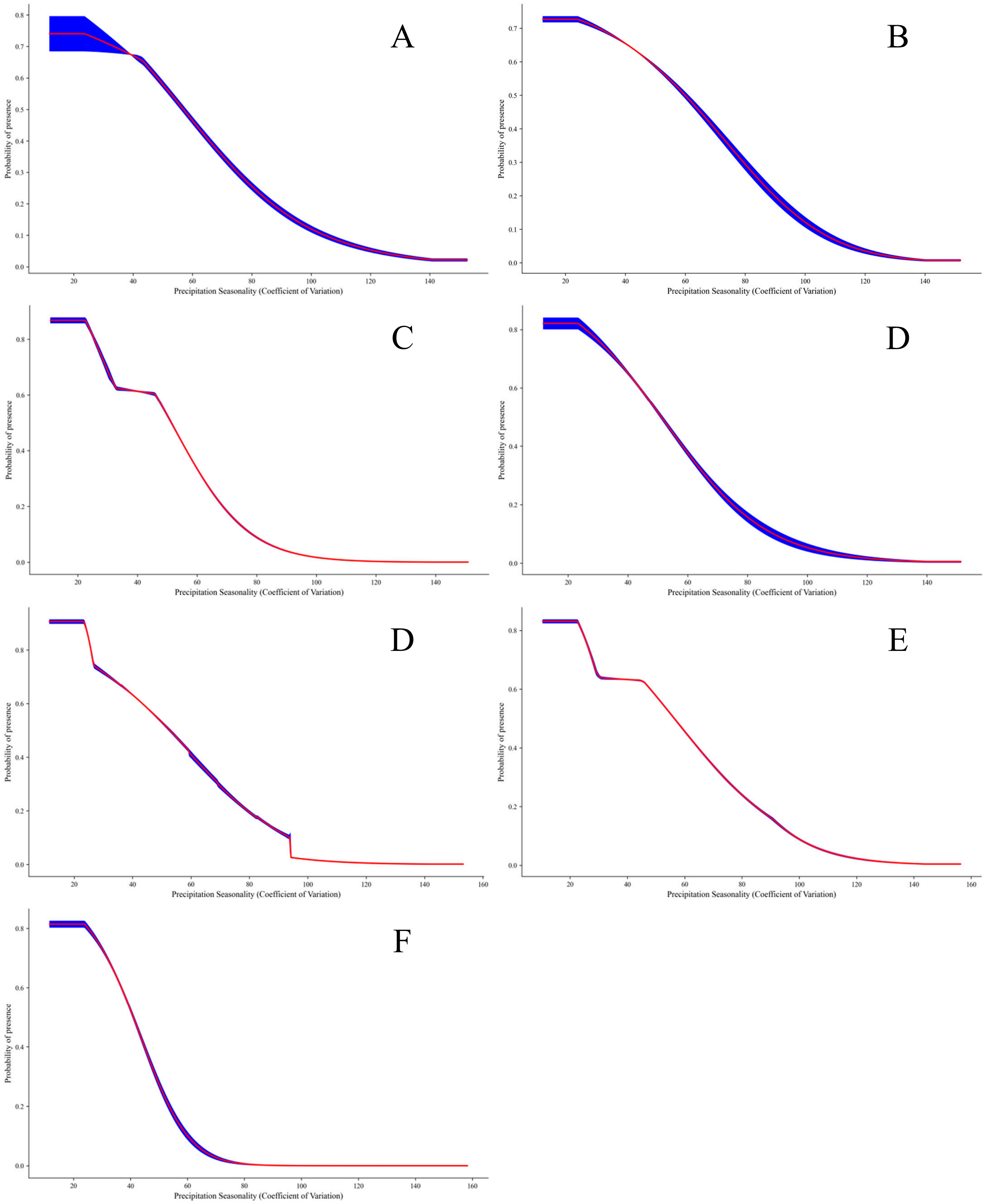

Supplementary Figure S3 Response curves of BIO15 for different falcon species

A *F.cherrug*; B *F.columbarius*; C *F.naumanni*; D *F.peregrinus*; E *F.subbuteo*; F *F.tinnunculus*; G *F.vespertinus*

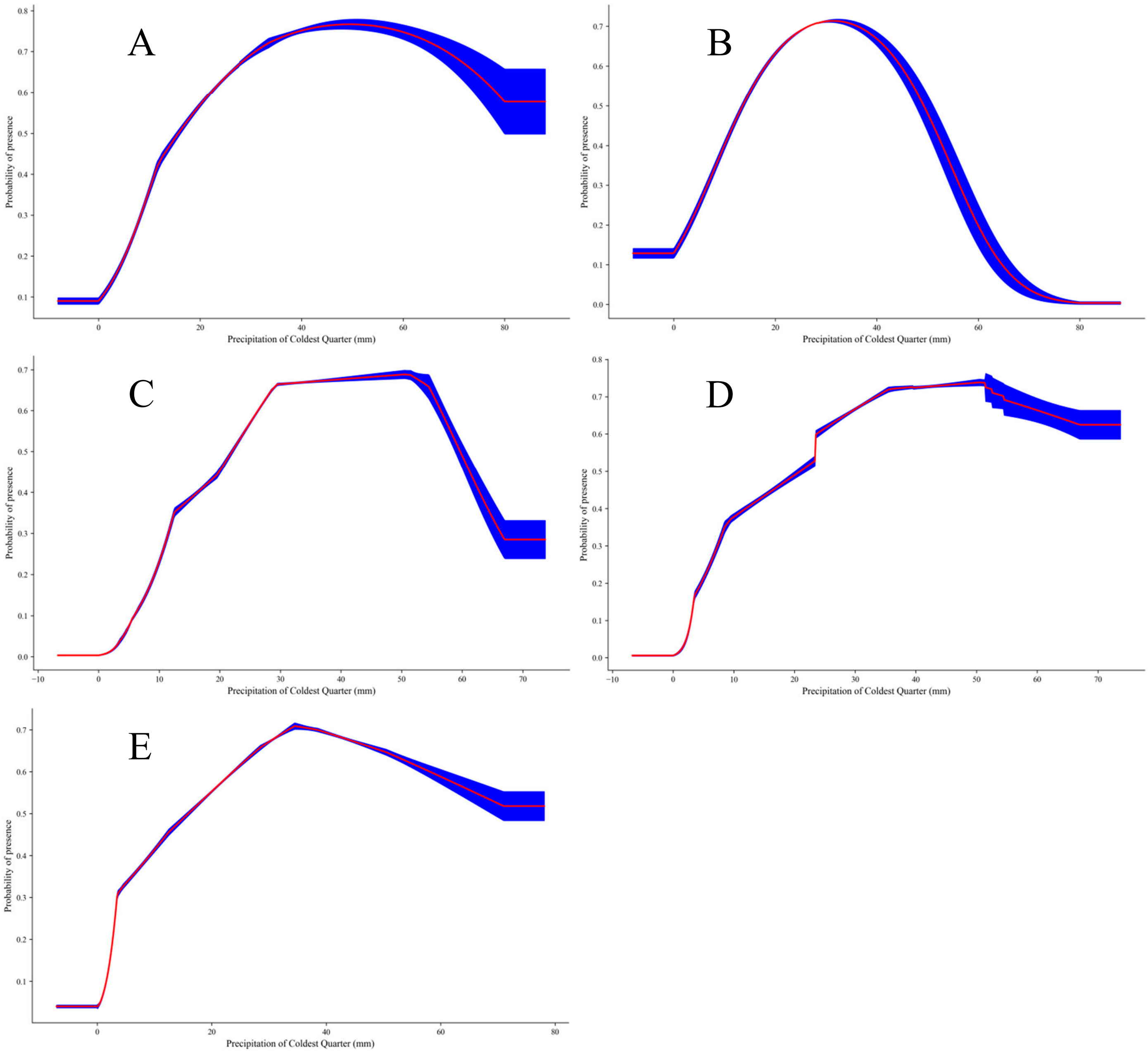

Supplementary Figure S4 Response curves of BIO19 for different falcon species
A *F.cherrug*; B *F.columbarius*; C *F.naumanni*; D *F.subbuteo*; E *F.tinnunculus*;
